# Antimicrobial susceptibility trends of WHO critical Gram-negative pathogens in Fijian Hospitals, 2016–2021

**DOI:** 10.1093/jacamr/dlag068

**Published:** 2026-05-07

**Authors:** Sakiusa C Baleivanualala, Vika Soqo, Shayal Smita, Sajnel Sharma, Swastika V Devi, Luse Delaitoga, Komal Maharaj, Sanjeshni Autar, Timoci Racolo, Numa Vera, Taina K Naivalu, Donald Wilson, John A Crump, James E Ussher

**Affiliations:** Department of Microbiology and Immunology, Faculty of Biomedical Sciences, University of Otago, Dunedin 9054, New Zealand; College of Medicine, Nursing and Health Science, Fiji National University, Suva, Fiji; Maurice Wilkins Centre for Molecular Biodiscovery, University of Auckland, Auckland 92019, New Zealand; Ministry of Health and Medical Services, Labasa Hospital, Labasa, Fiji; Lautoka Hospital, Aspen Medical, Lautoka, Fiji; Ministry of Health and Medical Services, Labasa Hospital, Labasa, Fiji; Ministry of Health and Medical Services, Colonial War Memorial Hospital, Suva, Fiji; College of Medicine, Nursing and Health Science, Fiji National University, Suva, Fiji; Lautoka Hospital, Aspen Medical, Lautoka, Fiji; Ministry of Health and Medical Services, Colonial War Memorial Hospital, Suva, Fiji; Ministry of Health and Medical Services, Colonial War Memorial Hospital, Suva, Fiji; Ministry of Health and Medical Services, Labasa Hospital, Labasa, Fiji; Ministry of Health and Medical Services, Colonial War Memorial Hospital, Suva, Fiji; College of Medicine, Nursing and Health Science, Fiji National University, Suva, Fiji; College of Medicine, Nursing and Health Science, Fiji National University, Suva, Fiji; Centre for International Health, University of Otago, Dunedin, New Zealand; Otago Global Health Institute, University of Otago, Dunedin 9054, New Zealand; Department of Microbiology and Immunology, Faculty of Biomedical Sciences, University of Otago, Dunedin 9054, New Zealand; Maurice Wilkins Centre for Molecular Biodiscovery, University of Auckland, Auckland 92019, New Zealand; Awanui Labs, Dunedin Hospital, Dunedin, New Zealand

## Abstract

**Introduction:**

Understanding antimicrobial susceptibility patterns is essential for guiding clinical management and informing surveillance strategies. This study aimed to analyse antimicrobial susceptibility trends of WHO-designated critical Gram-negative pathogens in Fijian hospitals.

**Methods:**

We conducted a retrospective study of antimicrobial susceptibility among *Acinetobacter baumannii*, *Pseudomonas aeruginosa*, *Escherichia coli*, and *Klebsiella pneumoniae* isolated from three hospitals in Fiji from 1 January 2016 to 31 December 2021. Data were stratified by hospital, hospital setting, and specimen type. Only the first isolate per patient was included. Chi-square tests and linear regression were used to assess group differences and temporal trends.

**Results:**

A total of 44 524 isolates were analysed: *K. pneumoniae* 17 016 (38.2%), *E. coli* 14 935 (33.5%), *P. aeruginosa* 6632 (14.9%), and *A. baumannii* 5941 (13.3%). Specimens included blood 4612 (10.4%), urine 13 369 (30.0%), and other types 26 543 (59.6%). Meropenem susceptibility declined significantly in *A. baumannii* (60.4% to 40.8%, *P* = 0.0004) and *P. aeruginosa* (100% to 40.8%, *P* = 0.005). Ceftriaxone susceptibility was low in *E. coli* (49.8%) and *K. pneumoniae* (32.7%). Meropenem susceptibility remained high in *K. pneumoniae* (96.6% in 2021) and *E. coli* (>80%). ESBL production was identified in 18.2% of *E. coli* and 37.6% of *K. pneumoniae*.

**Conclusions:**

These findings highlight substantial AMR challenges in Fijian hospitals, including declining carbapenem susceptibility and high ceftriaxone resistance, underscoring the need for strengthened antimicrobial stewardship, infection control, and surveillance systems.

## Introduction

Antimicrobial resistance (AMR) poses a major global health threat, undermining modern medicine and leading to increased mortality, prolonged hospital stays, and imposing a substantial economic burden.^1–3^ In 2019 alone, bacterial AMR was responsible for 1.27 million deaths and contributed to nearly 5 million fatalities globally.^2^ Persons living in low- and middle-income countries (LMICs), including those in the Pacific, bear a disproportionate burden of AMR due to limited healthcare infrastructure and a higher proportion of infectious diseases.^4,5^

*Acinetobacter baumannii*, *Pseudomonas aeruginosa*, and Enterobacterales, including *Escherichia coli* and *Klebsiella pneumoniae*, are designated by the WHO as pathogens of critical concern due to their ability to resist multiple, including last-resort antimicrobials.^6,7^ In Fiji, the absence of an AMR surveillance system exacerbates this challenge, impeding the comprehensive understanding of AMR percentages, trends, and patterns in the country. In this context, special studies using standard methods are needed to generate antibiogram data are essential to provide crucial insights into the percentages and trends of AMR, facilitating collaboration across healthcare facilities and countries.^7,8^

Fiji, an upper-middle-income country in the South Pacific with a population of under one million, plays a central role in the region’s economy and healthcare.^9^ Despite developing a national AMR plan,^10^ previous reports have underscored Fiji’s vulnerability to AMR pathogens.^7,11–13^ While antimicrobial stewardship initiatives promote evidence-based prescribing through guidelines that outline appropriate use, resistance-based management, and alignment with the national formulary, their effectiveness is limited by the lack of antimicrobial susceptibility data nationwide.

While the antimicrobial susceptibility of *A. baumannii*, *P. aeruginosa*, *E. coli*, and *K. pneumoniae* isolated from the Colonial War Memorial Hospital (CWMH), Suva, Viti Levu, Fiji, from 2019–2022 was recently published,^14^ there are no data from other parts of the country. To address this, our study aims to investigate trends of antimicrobial susceptibility of WHO critical pathogens isolated from clinical specimens at all three major hospitals in Fiji from 2016 through 2021. The findings aim to inform targeted interventions, strengthen antimicrobial stewardship, and lay the foundation for a national AMR surveillance framework in Fiji.

## Methods

### Study design and setting

We conducted a retrospective descriptive study analysing AST results of *A. baumannii*, *P. aeruginosa*, *E. coli*, and *K. pneumoniae* from microbiology laboratories of Fiji's three major hospitals: CWMH (523 beds), Lautoka Hospital (LTKH, 305 beds), and Labasa Hospital (LBSH, 180 beds) (Figure S1.0, available as Supplementary data at *JAC-AMR* Online). CWMH is Fiji’s largest tertiary hospital, serving the central and eastern divisions, which comprise the largest population in the country, and also serves as the main national referral centre. LTKH serves the western division, while LBSH serves the northern division. Together, these hospitals act as microbiology laboratory hubs, processing specimens referred from peripheral or outer health facilities nationwide. The study period spanned from 1 January 2016 to 31 December 2021. The selected pathogens were included due to their substantial role in nosocomial outbreaks within these hospitals, as reported in previous studies.^11,15,16^

### Microbiological testing

All hospitals inoculated specimens requiring culture and isolation onto appropriate culture media, with colonies assessed based on morphological characteristics. Gram staining was performed, with oxidase and indole tests conducted on Gram-negative bacteria (Tables S1a and b). Identification was completed using the Microbact^™^ Gram-Negative (GN) system (Oxoid, Hampshire, UK), with the Vitek 2 Compact system (bioMérieux, France) introduced at CWMH in 2020 for sterile site, urine, and critical care unit specimens. AST results were interpreted using CLSI M100 (2016) breakpoints for isolates tested between 2016 and 2018, and CLSI M100 (2019) breakpoints for isolates tested between 2019 and 2021.^17,18^ If isolates were resistant to ≥3 first-round agents, second-round agents were tested; meropenem and amikacin were reserved for isolates resistant to all second-round antimicrobials (Table S1c). ESBL production in Enterobacterales was confirmed using the double-disk synergy or combination disk method for isolates resistant to gentamicin or ceftriaxone and at least three first-line agents. Only ESBL-positive results were recorded. All laboratories participated in external quality assurance programs coordinated by the Royal College of Pathologists of Australasia (RCPA) in Australia and the Pacific Pathology Training Centre (PPTC) in New Zealand (Table S1d–f).

### Data collection and statistical analysis

AST data were collected from microbiology laboratory registers, except for CWMH (2019–2021), where the laboratory information system (LIS) was used. Cumulative antibiogram analysis followed CLSI guidelines, including only species with ≥30 isolates.^19^ For patients with multiple. In isolates, only the first isolate was included in polymicrobial specimens; all different species were included if deemed clinically significant. Intermediate results were classified as resistant, and cephalothin was excluded in line with the CLSI M100–2016 update.^17,20^

Data were categorized by hospital (CWMH, LTKH, LBSH), hospital setting (inpatient, outpatient, outer centres) by the time of collection, and specimen type (blood, urine, and other). In Fiji, ‘outer centres’ refer to peripheral health facilities such as health centres and nursing stations outside the main hospitals that collect specimens and refer them to CWMH, LTKH, and LBSH laboratories for microbiological testing. Other specimens included wound swabs, respiratory specimens, and catheter-derived specimens.

Percentages of susceptibility were calculated as the number of susceptible isolates divided by the total number tested. Temporal trends were assessed using linear regression, with the slope representing the rate of change. Group comparisons by hospital, setting, and specimen type used the Chi-square test. No adjustments were made for multiple comparisons; exact *P*-values are reported in Tables S2–S5. Statistical analyses and visualizations were performed using GraphPad Prism v9.5.0.

## Results

A total of 44 524 isolates of targeted organisms were identified from the microbiology records of the three hospitals from 1 January 2016 through 31 December 2021. Of these, 26 543 (59.6%) were from other specimen types. Among the other specimens, respiratory samples accounted for 8567 (32.3% of other specimens; 19.2% of total isolates), with the remainder comprising wound swabs and catheter-derived specimens. Urine comprised 13 369 (30.0%), and 4612 (10.4%) from blood. Moreover, 29 708 (66.7%) reports were associated with hospitalized patients. Of the identified target organisms, 17 016 (38.2%) were *K. pneumoniae*, 14 935 (33.5%) were *E. coli*, 6632 (14.9%) were *P. aeruginosa*, and 5941 (13.3%) were *A. baumannii*.

### Acinetobacter baumannii

Of the 5941 *A. baumannii* isolates reported from the three hospital microbiology laboratories, 4440 (74.7%) were from inpatients, 886 (14.9%) from outer centres, and 615 (10.4%) from outpatients. The most common specimen source was other specimens (4670; 78.6%), followed by urine (683; 11.5%) and blood (588; 9.9%). By hospital, 2620 (44.1%) isolates were from CWMH, 2068 (33.8%) from LTKH, and 1253 (21.1%) from LBSH.

### *A. baumannii* antimicrobial susceptibility by year

**Figure 1.**
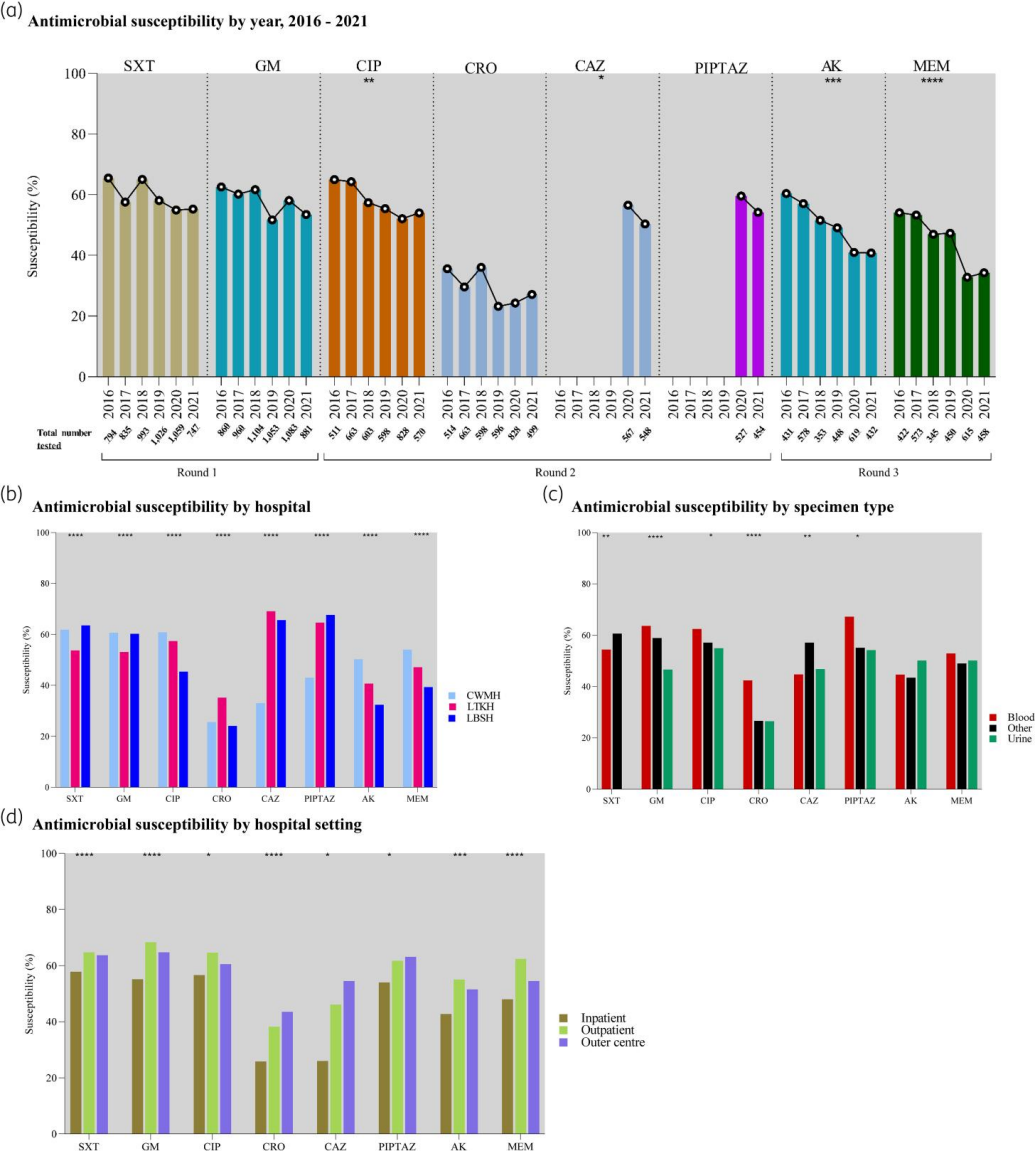
Antimicrobial susceptibility of *Acinetobacter baumannii* complex, Fiji, 2016–2021. SXT, trimethoprim-sulfamethoxazole; GM, gentamicin; CIP, ciprofloxacin; CRO, ceftriaxone; CAZ, ceftazidime; PIP, piperacillin; PIPTAZ, piperacillin-tazobactam; AK, amikacin; MEM, meropenem. Asterisks indicate that the change was statistically significant. *P < 0.05; **P < 0.01; ***P < 0.001; ****P < 0.0001 with more asterisks indicating stronger evidence. Note: Differences in susceptibility percentages were modest despite statistical significance.

Among *A. baumannii* isolates, susceptibility to several antimicrobials declined significantly over the study period (Figure 1a; Table S2a). Ciprofloxacin susceptibility declined from 65.0% (332/511) in 2016 to 54.0% (308/570) in 2021, declining by 2.7% per year (95% CI −4.2 to −1.1; *P* = 0.01). Ceftazidime susceptibility declined from 56.6% (321/567) in 2020 to 50.4% (276/548) in 2021, declining by 6.2% per year (95% CI −11.8 to −0.1; *P* = 0.04). Amikacin susceptibility declined from 54.1% (233/431) to 34.3% (148/432), at 7.1% per year (95% CI −12.1 to −2.1; *P* = 0.01). For meropenem, cumulative susceptibility across the study period was 49.6% (1420/2863). Meropenem susceptibility declined from 60.4% (255/422) to 40.8% (187/458), at 4.3% per year (95% CI −5.4 to −3.1; *P* = 0.0004). Declining trends were also observed for gentamicin, piperacillin-tazobactam, and ceftriaxone, although not statistically significant.

### *A. baumannii* antimicrobial susceptibility in hospitals

LBSH had a lower percentage of *A. baumannii* susceptibility to several antimicrobials compared with CWMH and LTKH. Ciprofloxacin susceptibility at LBSH was 45.4% (215/474), lower than LTKH (57.4%, 791/1377) and CWMH (60.8%, 1168/1922). Amikacin susceptibility at LBSH was 32.4% (132/408), compared with 40.7% (399/981) at LTKH and 50.2% (739/1472) at CWMH. Ceftriaxone susceptibility was lowest at LBSH (24.1%, 116/481), followed by CWMH (25.6%, 478/1866) and LTKH (35.2%, 475/1351).

LTKH *A. baumannii* showed a lower susceptibility to trimethoprim-sulfamethoxazole (53.7%, 997/1856) compared to CWMH (61.8%, 1547/2505) and LBSH (63.5%, 694/1093). Gentamicin susceptibility at LTKH was 53.1% (1098/2068), lower than LBSH (60.2%, 754/1253) and CWMH (60.6%, 1589/2620). The percentage of ceftazidime susceptibility was lowest at CWMH (33.0%, 151/457), compared to LBSH (65.6%, 164/250) and LTKH (69.1%, 282/408). Similarly, piperacillin-tazobactam susceptibility was lowest at CWMH (53.1%, 197/458), compared to LTKH (64.6%, 246/381) and LBSH (67.6%, 171/253). All comparisons were statistically significant (*P* < 0.0001; see Figure 1b and Table S2b).

### *A. baumannii* susceptibility by specimen type

Isolates of *A. baumannii* from urine exhibited a lower percentage of susceptibility to most antimicrobials compared to isolates from blood (Figure 1c; Table S2c). The percentage of gentamicin susceptibility was 46.6% (318/683) in urine versus 63.6% (374/588) in blood; ciprofloxacin was 54.9% (254/463) in urine versus 62.4% (347/556) in blood; ceftriaxone was 26.5% (125/472) in urine versus 42.4% (233/550) in blood; and piperacillin-tazobactam was 54.2% (110/203) in urine compared to 67.2% (80/119) in blood. Other specimen types showed resistant values for these agents. In contrast, trimethoprim-sulfamethoxazole and ceftazidime susceptibility were lowest in blood, compared with other specimen types, at 54.4% (357/656) and 44.7% (68/152), respectively.

### *A. baumannii* antimicrobial susceptibility by hospital settings

*A. baumannii* isolated from inpatients showed the lowest susceptibility to all antimicrobials tested (Figure 1d; Table S2d). The percentage of susceptibility was as follows: trimethoprim-sulfamethoxazole (57.8%, 2368/4097), ciprofloxacin (56.6%, 1747/3084), gentamicin (55.1%, 2448/4440), piperacillin-tazobactam (54.0%, 438/811), ceftazidime (51.0%, 423/830), meropenem (48.0%, 1145/2385), amikacin (42.7%, 1021/2390), and ceftriaxone (25.8%, 770/2979)

### Pseudomonas aeruginosa

Of the 6632 *P. aeruginosa* isolates reported from the three hospital microbiology laboratories, 4382 (66.1%) were from inpatients, 1159 (17.5%) from outer centres, and 1091 (16.5%) from outpatients. Other specimens were the most common specimen source (5532; 83.4%), followed by urine (803; 12.1%) and blood (297; 4.5%). By hospital, 2596 (39.1%) of isolates were from CWMH, 2083 (31.4%) from LTKH, and 1953 (29.4%) from LBSH.

### Antimicrobial susceptibility of *P. aeruginosa* by year

Gentamicin susceptibility declined from 75.9% (663/873) in 2016 to 70.1% (653/931) in 2021, declining at 1.3% per year (95% CI −1.8 to −0.8; *P* = 0.002). Ceftazidime susceptibility declined from 91.9% (799/869) to 88.4% (731/827), at 0.7% per year (95% CI −1.3 to −0.2; *P* = 0.02). Piperacillin susceptibility declined from 85.1% (744/874) to 74.7% (186/249), at 1.6% per year (95% CI −3.1 to −0.2; *P* = 0.007).

**Figure 2.**
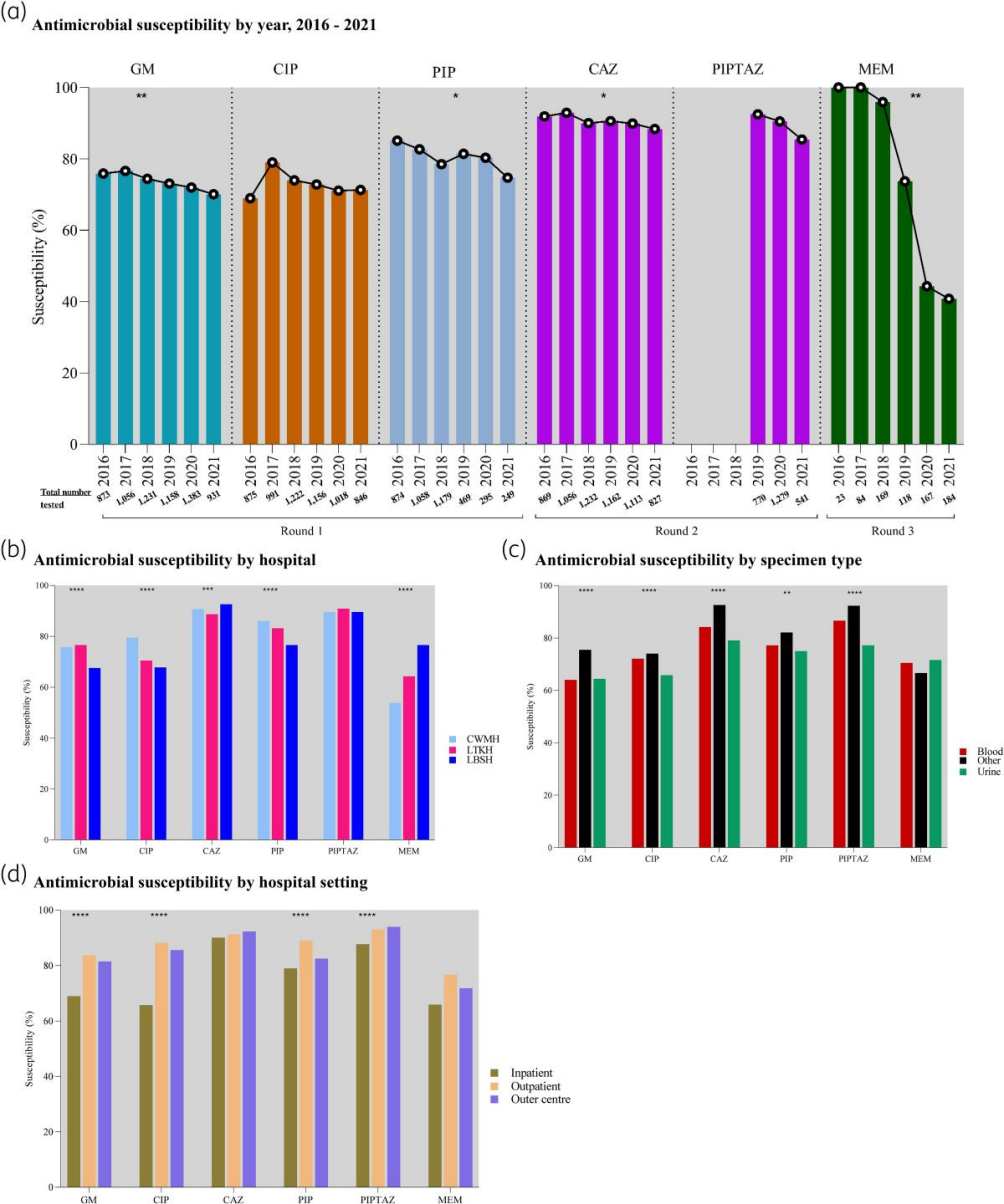
Antimicrobial susceptibility of *P. aeruginosa*, Fiji, 2016–2021. GM, gentamicin; CIP, ciprofloxacin; CAZ, ceftazidime; PIP, piperacillin; PIPTAZ, piperacillin-tazobactam; MEM, meropenem. Asterisks indicate that the change was statistically significant. *P < 0.05; **P < 0.01; ***P < 0.001; ****P < 0.0001 with more asterisks indicating stronger evidence. Note: Differences in susceptibility percentages were modest despite statistical significance.

Meropenem susceptibility was assessed in 745 (11.2%) of the 6660 isolates, with an overall susceptibility of 67.8% (505/745). Meropenem susceptibility declined sharply from 100.0% (23/23) in 2016 to 40.8% (75/184) in 2021, at 13.9% per year (95% CI −20.8 to −6.9; *P* = 0.005). Changes for ciprofloxacin and piperacillin-tazobactam were not statistically significant. (Figure 2a; Table S3a).

### *P. aeruginosa* susceptibility in hospitals

When comparing *P. aeruginosa* susceptibility across hospitals (Figure 2b; Table S3b), the percentage of gentamicin susceptibility was 67.6% (1320/1953) at LBSH, 75.8% (1968/2596) at CWMH, and 76.6% (1595/2083) at LTKH.

For piperacillin, LBSH had 76.6% (1306/1706) susceptibility, lower than LTKH (83.2%, 1101/1323) and CWMH (86.1%, 943/1095). The percentage of ciprofloxacin susceptibility was also lower at LBSH (67.8%, 1281/1890) compared to LTKH (70.5%, 1359/1928) and CWMH (79.5%, 1821/2290). In contrast, LTKH showed a lower percentage of ceftazidime susceptibility (88.7%, 1749/1972) than CWMH (90.7%, 2192/2416) and LBSH (92.6%, 1733/1871). Meropenem susceptibility was lowest at CWMH (53.8%, 98/182), compared to LTKH (64.3%, 126/196) and LBSH (76.6%, 281/367).

### *P. aeruginosa* susceptibility by specimen types

*P. aeruginosa* isolates from urine exhibited lower susceptibility to most tested antimicrobials compared to other specimen types (Figure 2c; Table S3c). The percentage of susceptibility in urine versus other specimens was: ceftazidime 79.1% (561/709) versus 92.6% (4874/5266), piperacillin 54.9% (282/463) versus 82.1% (2946/3590), ciprofloxacin 65.8% (445/676) versus 74.0% (3809/5145), and piperacillin-tazobactam 77.2% (275/356) versus 92.3% (1974/2139). For these antimicrobials, susceptibility in blood isolates fell between those of urine and other specimen types. Gentamicin susceptibility was comparable in urine (64.4%, 517/803) and blood (64.0%, 190/297), but higher in other specimens (75.5%, 4176/5532).

### *P. aeruginosa* susceptibility by hospital setting

*P. aeruginosa* isolates from hospitalized patients showed the lowest susceptibility to all antimicrobials tested (Figure 2d; Table S3d). Among inpatients, susceptibility was 69.0% (3025/4382) for gentamicin, 65.7% (2618/3987) for ciprofloxacin, 79.0% (2184/2766) for piperacillin, and 87.7% (1429/1629) for piperacillin-tazobactam.

### Escherichia coli

Of the 14 935 *E. coli* isolates reported from the three hospital microbiology laboratories, 9037 (60.5%) were from inpatients, 3016 (20.2%) from outpatients, and 2882 (19.3%) from outer centres. Urine was the most common specimen source (6448: 43.2%), followed by other specimens (6305; 42.2%) and blood (2182; 14.6%). By hospital, 6364 (42.6%) of isolates were from LTKH, 5560 (37.2%) from CWMH, and 3011 (20.2%) from LBSH.

### Susceptibility by year

**Figure 3.**
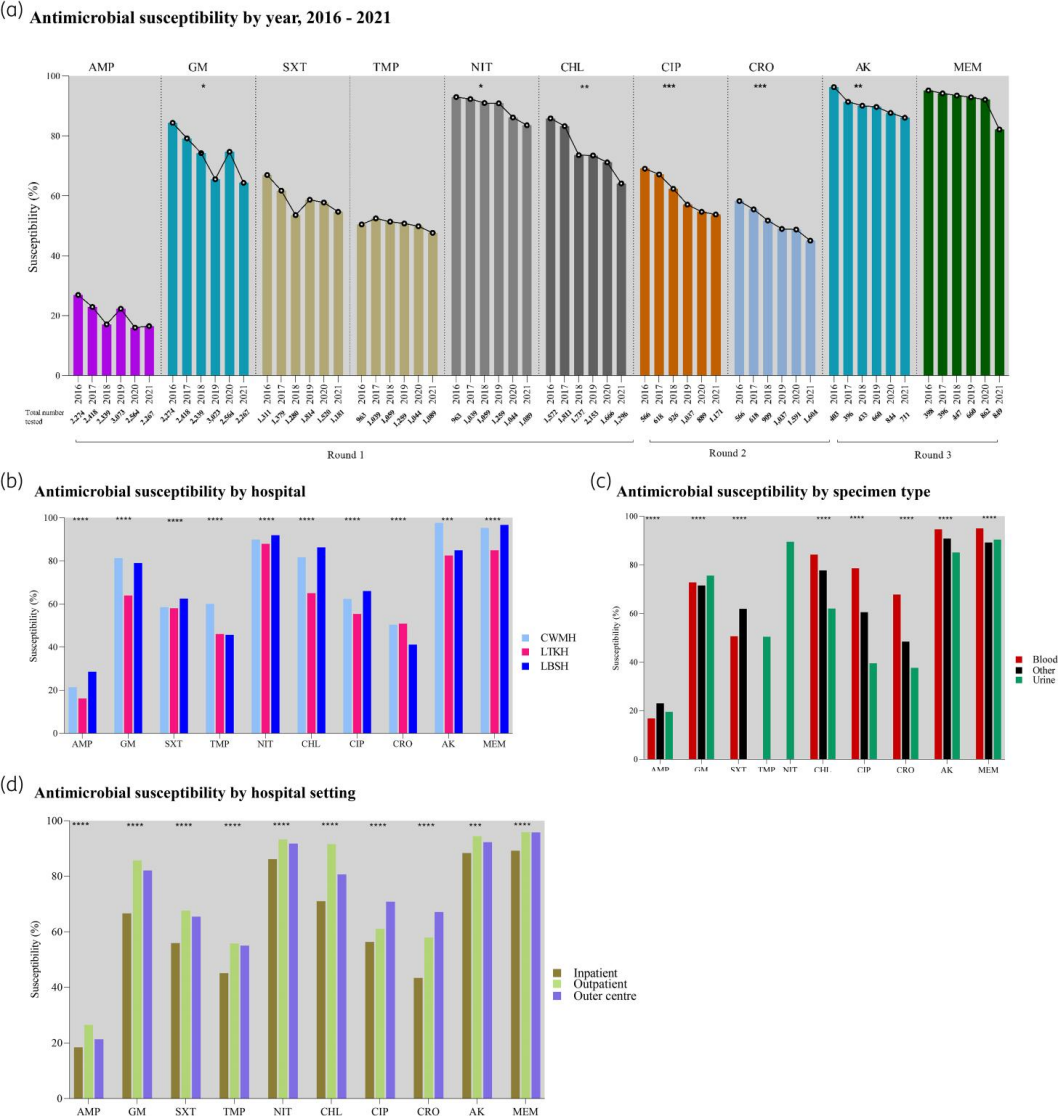
Antimicrobial susceptibility of *Escherichia coli*, Fiji, 2016–2021. AMP, ampicillin; GM, gentamicin; SXT, trimethoprim-sulfamethoxazole; TMP, trimethoprim, NIT, nitrofurantoin; CHL, chloramphenicol; CIP, ciprofloxacin; CRO, ceftriaxone; AK, amikacin; MEM, meropenem. Asterisks indicate that the change was statistically significant. *P < 0.05; **P < 0.01; ***P < 0.001; ****P < 0.0001. Note: Differences in susceptibility percentages were modest despite statistical significance.

All antimicrobials tested against *E. coli* showed declining susceptibility trends from 2016 to 2021 (Figure 3a; Table S4a), with statistically significant reductions for several agents. Gentamicin susceptibility declined from 84.4% (1920/2274) in 2016 to 64.4% (1460/2267) in 2021, declining at 3.5% points per year (95% CI −6.6 to −0.4; *P* = 0.03). Nitrofurantoin susceptibility declined from 93.0% (896/963) in 2016 to 82.7% (901/1089) in 2021, at 1.9 points per year (95% CI −2.9 to −0.9; *P* = 0.01). Chloramphenicol fell from 85.9% (1351/1572) to 64.2% (832/1296), at 4.1 points per year (95% CI −5.7 to −2.6; *P* = 0.002). Ciprofloxacin declined from 69.1% (391/566) to 53.8% (630/1171), at 3.4% points per year (95% CI −4.4 to −2.4; *P* = 0.0007). Ceftriaxone fell from 58.3% (330/566) to 45.1% (724/1604), at 2.5% points per year (95% CI −3.2 to −1.9; *P* = 0.0004). Amikacin declined from 96.3% (388/403) to 86.1% (612/711), at 1.8% points per year (95% CI −2.6 to −0.1; *P* = 0.004). Changes in ampicillin, trimethoprim-sulfamethoxazole, trimethoprim, and meropenem were not statistically significant. Overall, meropenem maintained high cumulative susceptibility at 90.7% (3275/3612).

### Susceptibility by hospitals

LTKH consistently showed lower *E. coli* susceptibility to several antimicrobials compared to CWMH and LBSH (Figure 3b; Table S4b). Ampicillin susceptibility was lowest at LTKH (16.2%, 1030/6364) versus 21.4% (1190/5560) at CWMH and 28.6% (860/3011) at LBSH. Gentamicin susceptibility was also lower at LTKH (63.9%, 4067/6364) compared to 81.3% at CWMH and 79.0% at LBSH. Similar trends were observed for chloramphenicol (65.0% at LTKH versus 81.7% at CWMH and 86.3% at LBSH), ciprofloxacin (55.4% versus 62.4% and 66.0%), and amikacin (82.5% versus 97.6% and 84.9%). Meropenem susceptibility was 84.9% at LTKH, compared to 95.3% at CWMH and 96.7% at LBSH. In contrast, LBSH had the lowest susceptibility to trimethoprim (45.7%) and ceftriaxone (41.2%), compared to 46.1% and 50.9% at LTKH, and 60.0% and 50.4% at CWMH, respectively.

### Susceptibility by specimen types

*E. coli* isolated from urine showed lower susceptibility to most antimicrobials compared to isolates from other specimens and blood (Figure 3c; Table S4c). Chloramphenicol susceptibility in urine was 62.0% (1378/2222) versus 77.7% (4580/5897) in other specimens. Ciprofloxacin susceptibility was 39.5% (601/1521) in urine compared to 60.5% (1353/2238), and ceftriaxone was 37.6% (778/2070) versus 48.4% (1271/2626). Amikacin susceptibility was slightly lower in urine (85.1%, 1041/1223) than in other specimens (90.8%, 1434/1580).

In contrast, isolates from blood showed the highest susceptibility to most antimicrobials: gentamicin 72.8% (1588/2182), chloramphenicol 84.2% (1781/2116), ciprofloxacin 78.6% (1138/1448), ceftriaxone 67.8% (1104/1629), amikacin 94.6% (609/644), and meropenem 95.0% (614/646).

### Susceptibility by hospital setting

*E. coli* isolates from hospitalized patients demonstrated the lowest susceptibility across all antimicrobials tested (Figure 3d; Table S4d). Susceptibility percentages among inpatient isolates were ampicillin 18.4% (1667/9037), gentamicin 66.6% (6023/9037), trimethoprim-sulfamethoxazole 55.9% (3329/5952), trimethoprim 45.1% (1417/3141), nitrofurantoin 86.1% (2705/3141), chloramphenicol 71.0% (4901/6898), ciprofloxacin 56.3% (2079/3696), ceftriaxone 43.4% (1866/4300), amikacin 88.3% (2301/2607), and meropenem 89.1% (2471/2773).

### ESBL producing *E. coli*

Of the 14 935 *E. coli* isolates, 18.2% (2717/14 935) were identified as ESBL producers (Table S4e). Of these, 43.9% were from urine, 39.6% from other specimen types, and 16.5% from blood. The percentage of ESBL-producing *E. coli* significantly increased from 11.8% (269/2274) in 2016 to 25.8% (586/2267) in 2021, at a rate of 2.7% points per year (95% CI 0.7 to 4.8; *P* = 0.02).

By hospital, ESBL percentage was highest at CWMH (21.5%, 1194/5560), followed by LTKH (18.9%, 1200/6364) and LBSH (10.7%, 323/3011). ESBLs were more frequently detected in hospitalized patients (22.6%, 2040/9037), compared to outpatients (10.9%, 329/3016) and patients from outer centres (12.1%, 348/2882).

### Klebsiella pneumoniae

Of the 17 016 *K. pneumoniae* isolates reported from the three hospital microbiology laboratories, 11 849 (69.6%) were collected from inpatients, 2685 (15.8%) from outer centres, and 2482 (14.6%) from outpatients. The most common specimen source was ‘other specimens’ (10 036; 59.0%), followed by urine (5435; 31.9%) and blood (1545; 9.1%). By site, 7131 (41.9%) of isolates were from CWMH, 5833 (34.3%) from LTKH, and 4052 (23.8%) from LBSH.

### Susceptibility by year

**Figure 4.**
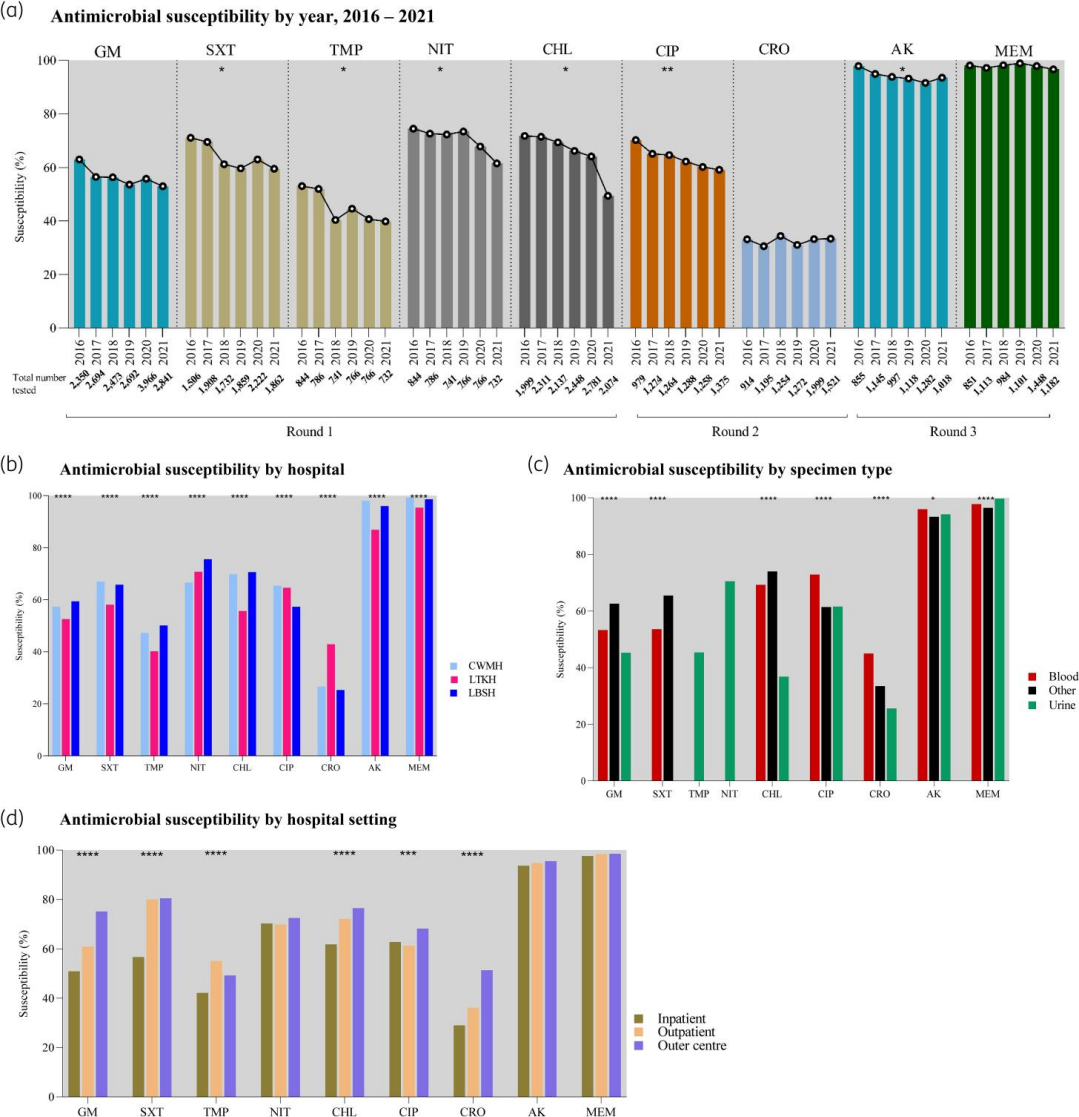
Antimicrobial susceptibility of *Klebsiella pneumoniae*, Fiji, 2016–2021. GM, gentamicin; SXT, trimethoprim-sulfamethoxazole; TMP, trimethoprim, NIT, nitrofurantoin, CHL, chloramphenicol; CIP, ciprofloxacin; CRO, ceftriaxone; AK, amikacin; MEM, meropenem. Asterisks indicate that the change was statistically significant. *P < 0.05; **P < 0.01; ***P < 0.001; ****P < 0.0001. Note: Differences in susceptibility percentages were modest despite statistical significance.

Among *K. pneumoniae* isolates, susceptibility to most antimicrobials declined over the study period (Figure 4a; Table S5a). Trimethoprim-sulfamethoxazole susceptibility fell significantly from 71.1% (1071/1506) in 2016 to 59.5% (1108/1862) in 2021, declining by 2.3% points per year (95% CI −4.3 to −0.2; *P* = 0.04). Trimethoprim declined from 53.1% (448/844) to 39.9% (292/732), at 2.7% points per year (95% CI −5.1 to −0.4; *P* = 0.03). Nitrofurantoin declined from 74.5% (629/844) to 61.5% (450/732), at 2.2% points per year (95% CI −4.1 to −0.3; *P* = 0.03). Chloramphenicol declined from 71.8% (1435/1999) to 49.4% (1025/2074), at 3.9% points per year (95% CI −7.0 to −0.9; *P* = 0.02). Ciprofloxacin declined from 70.2% (687/979) to 59.1% (812/1375), at 2.1% points per year (95% CI −2.8 to −1.3; *P* = 0.002). Amikacin fell from 97.9% (837/855) to 93.5% (952/1018), at 0.9% points per year (95% CI −1.8 to −0.02; *P* = 0.047). Declining trends in gentamicin and meropenem were not statistically significant, and ceftriaxone remained stable. Cumulative meropenem susceptibility among *K. pneumoniae* isolates was 90.7% (3275/3612).

### Susceptibility by hospital

*K. pneumoniae* isolates from LTKH showed lower susceptibility to several antimicrobials compared to CWMH and LBSH (Figure 4b; Table S5b). Gentamicin susceptibility was lowest at LTKH (52.6%, 3069/5833), compared to CWMH (57.3%, 4087/7131) and LBSH (59.4%, 2407/4052). Trimethoprim-sulfamethoxazole susceptibility at LTKH was 58.1% (2047/3525), lower than CWMH (67.0%, 3066/4579) and LBSH (65.8%, 1963/2985). For trimethoprim, LTKH had 40.2% (692/1722), compared to 47.2% (811/1717) at CWMH and 50.1% (599/1196) at LBSH. Chloramphenicol susceptibility was 55.7% (2477/4449) at LTKH, versus 69.8% (4033/5774) at CWMH and 70.6% (2491/3527) at LBSH. Amikacin susceptibility was also lower at LTKH (86.9%, 1803/2074) compared to LBSH (96.0%, 1399/1457) and CWMH (98.0%, 2825/2884). In contrast, LBSH had the lowest susceptibility to ciprofloxacin (57.3%, 984/1718) and ceftriaxone (25.3%, 434/1718). Meropenem susceptibility remained high across all hospitals: LTKH (95.4%, 2178/2282), LBSH (98.6%, 1437/1457), and CWMH (99.2%, 2916/2940).

### Susceptibility by specimen types

*K. pneumoniae* susceptibility varied significantly by specimen type (Figure 4c; Table S5c). Urine isolates had the lowest susceptibility to gentamicin (45.3%, 2462/5435), chloramphenicol (36.8%, 1096/2978), and ceftriaxone (25.6%, 661/2579). Ciprofloxacin susceptibility was similarly low in both urine (61.6%, 1549/2515) and other specimens (61.4%, 2330/3793). Blood isolates showed the lowest susceptibility to trimethoprim-sulfamethoxazole (53.6%, 828/1545). Among urine isolates, nitrofurantoin demonstrated relatively high susceptibility (70.5%, 3267/4635), while trimethoprim remained low at 45.4% (2102/4635). Across all specimen types, susceptibility to amikacin and meropenem remained consistently high, both exceeding 93%.

### Susceptibility by hospital setting

*K. pneumoniae* isolates from hospitalized patients showed the lowest susceptibility to most antimicrobials (Figure 4d; Table S5d). Among inpatients, susceptibility was 50.9% for gentamicin (6035/11 849), 56.7% for trimethoprim-sulfamethoxazole (4387/7741), 42.2% for trimethoprim (1369/3245), 61.8% for chloramphenicol (6053/9801), and 29.0% for ceftriaxone (1803/6216). Ciprofloxacin susceptibility was lowest among outpatients at 61.3% (423/690), compared to 62.8% (3730/5942) for inpatients and 68.2% (550/806) for

### ESBL producing *K. pneumoniae*

Of the 17 016 *K. pneumoniae isolates*, 6393 (37.6%) were identified as ESBL producers (Table S5e). These were most frequently isolated from other specimens (50.1%, 3200/6393), followed by urine (38.2%, 2443/6393) and blood (11.7%, 750/6393). ESBL percentage varied over time, peaking at 48.7% (1311/2692) in 2019 but showing no consistent trend, with 33.0% (775/2350) in 2016 and 33.9% (964/2841) in 2021. By hospital, ESBL percentages were highest at LBSH (38.8%, 1571/4052), followed by CWMH (38.0%, 2708/7131) and LTKH (36.2%, 2114/5833). ESBLs were more common among hospitalized patients (44.4%, 5262/11 849) compared to outpatients (23.3%, 579/2482) and patients from other centres (20.6%, 552/2685).

## Discussion

We demonstrated a high and increasing percentage of AMR among WHO critical Gram-negative pathogens, including *A. baumannii*, *P. aeruginosa*, *E. coli*, and *K. pneumoniae*, isolated from clinical specimens in Fiji from 2016 through 2021. Resistance was observed to both commonly used and last-line antimicrobials, such as meropenem. Highest resistance was observed among hospitalized setting, highlighting the increased risk of nosocomial-associated AMR infections. This is particularly concerning as a recent study at the CWMH reported a decline in meropenem susceptibility through 2022, alongside the emergence of carbapenemase-producing organisms, highlighting the worsening AMR landscape.^14^ Although CWMH had a slightly higher susceptibility percentage than LBSH and LTKH, all hospitals experienced declining trends, underscoring the need for national-level interventions.

*Acinetobacter baumannii* showed widespread resistance, with susceptibility below 70% for all tested antimicrobials. Of great concern is the persistence of carbapenem-resistant *A. baumannii* (CR*Ab*), associated with prolonged outbreaks in Fiji hospitals since 2016.^11,15^ Meropenem resistance has been predominantly mediated by *bla*_OXA-23_.^15^ Of note, Fiji’s cumulative meropenem susceptibility (49.6%) is lower than that reported in French Polynesia (58.3%),^21^ New Caledonia (75.2%),^22^ Australia (96.6%),^23^ and New Zealand (95.6%),^24^ though comparable to Samoa (40%).^7^ However, only 46.4% of *A. baumannii* isolates were tested for meropenem due to the tiered testing strategy, which may have underestimated meropenem susceptibility. Declining susceptibility trends were also observed for ciprofloxacin, ceftriaxone, and amikacin, particularly in isolates from LBSH, which showed the lowest overall susceptibility. Inpatient isolates consistently exhibited lower susceptibility. Interestingly, meropenem susceptibility among blood isolates was only 52.9%, raising concerns about empiric use without AST confirmation. While Fiji's guidelines recommend reserving meropenem for bloodstream infections or when no alternatives are available, the relatively low susceptibility (52.9%) of blood isolates highlights the significant risk of treatment failure and underscores the need for combination therapy or rapid susceptibility testing before meropenem is initiated as monotherapy.

*Pseudomonas aeruginosa* is a known cause of healthcare-associated infections in Fiji, often linked to medical devices and moist environments.^25^ It poses a major challenge due to its adaptive resistance mechanisms and rapid mutation rates.^26^ Of note, meropenem susceptibility declined significantly from 100% in 2016 to 40.8% in 2021, a 59.2% drop. Cumulative meropenem susceptibility was markedly lower than in other Pacific Island nations such as Samoa, Kiribati, and the Cook Islands, where rates range from 91.7% to 98.3%.^7^ However, only 11.2% of 6, 632 *P. aeruginosa* isolates were tested for meropenem, likely underestimating susceptibility. Susceptibility to antipseudomonal antimicrobials such as gentamicin, ciprofloxacin, and piperacillin was also <80% across all hospitals. CWMH reported the lowest meropenem susceptibility, while LBSH showed the lowest rates for other antimicrobials. Inpatient isolates remained the most resistant.

Both *E. coli* and *K. pneumoniae* demonstrated declining susceptibility to commonly used antimicrobials. *E. coli*, a leading cause of urinary tract infections,^27,28^ showed a 10%–22% reduction in susceptibility to gentamicin, nitrofurantoin, and chloramphenicol. Ciprofloxacin, ceftriaxone, and the last-line agent amikacin also declined by 13%–22%. *K. pneumoniae* showed similar trends, with susceptibility to first-line agents falling by 11%–23%, ciprofloxacin by 11%, and amikacin by 4%. Of note, trimethoprim susceptibility was <50% for both pathogens, suggesting it is no longer suitable for empiric treatment. Nitrofurantoin remained more effective (89.5% in *E. coli*, 70.5% in *K. pneumoniae*), though Fiji’s rates were lower than Samoa (97%) and Tonga (92.6%).^7^ Of great concern was the rise in ceftriaxone resistance among Enterobacterales. Only 49.8% of 6325 *E. coli* and 32.7% of 8155 *K. pneumoniae* tested were susceptible. Even if untested isolates are assumed susceptible, median resistance estimates remain high (21.3% and 32.3%, respectively). Compared to WHO GLASS data from 76 countries, Fiji’s ceftriaxone resistance rates are significantly higher: 50.2% versus 42.0% for *E. coli* and 67.3% versus 35.0% for *K. pneumoniae*.^29^ Despite the link between ceftriaxone resistance and ESBL production, ESBL testing was mostly performed for gentamicin-resistant isolates, identifying ESBLs in only 18.2% of *E. coli* and 37.6% of *K. pneumoniae*. Other mechanisms, such as *AmpC* β-lactamase or carbapenemase production, may also contribute, although meropenem resistance remains low at 9.3% and 2.2% for *E. coli* and *K. pneumoniae,* respectively. Of note, recent studies in Fiji identified ESBL-producing *E. coli* and *K. pneumoniae* carrying *bla*_CTX-M-15_ gene in both hospital and community settings, suggesting that transmission may be occurring beyond hospital settings.^16,30^ Interestingly, cephalothin remained in use across all three laboratories for first-line testing of Enterobacterales, despite its removal from CLSI M100 guidelines. Its continued use highlights Fiji’s need to update AST panels to align with current standards and ensure data comparability. While susceptibility to last-line agents like amikacin and meropenem remains high (90.7%–97.8%), the risk of increasing resistance persists, especially in the context of poor infection prevention and control (IPC), overcrowding, inconsistent PPE use, and disrupted antimicrobial supply chains.^15,31,32^

While this study provides valuable insights into antimicrobial susceptibility trends in Fiji, several limitations should be acknowledged. As a retrospective observational study, causal relationships cannot be established, and findings should be interpreted accordingly. First, stock shortages and reagent availability constrained the scope of AST. Second, the tiered testing approach may have biased susceptibility estimates. Third, inconsistent application of CLSI breakpoints across laboratories may have affected comparability. Fourth, the absence of EQA reports at LTKH for four years raises concerns about diagnostic reliability. Fifth, lack of clinical metadata prevented classification of infections as community or hospital acquired. Lastly, the COVID-19 pandemic may have introduced bias due to changes in healthcare access and antimicrobial prescribing. These factors may have introduced both selection bias and information bias and should be considered when interpreting the findings.

In conclusion, the study highlights the growing burden of AMR among WHO critical Gram-negative pathogens in Fiji, with rising resistance to both first line and last-resort antimicrobials. These findings call for urgent action to strengthen antimicrobial stewardship, revise treatment and AST guidelines, and implement a national AMR surveillance system. Standardized AST, routine metadata collection, and molecular tools such as whole-genome sequencing will be critical for targeted interventions. Addressing diagnostic gaps, improving IPC, and enhancing regional collaboration are essential to preserve antimicrobial effectiveness and protect public health in Fiji.

## Supplementary Material

dlag068_Supplementary_Data

## Data Availability

All data used in this analysis are available in the supplementary.

## Supplementary data

Figure S1.0 and Tables S1 to S4 are available as Supplementary data at *JAC-AMR* Online.
