# Supplementary material for: Antimicrobial susceptibility trends of WHO critical Gram-negative pathogens in Fijian Hospitals, 2016–2021
Source: JAC Antimicrob Resist. 2026 May 7;8(3):dlag068. doi: 10.1093/jacamr/dlag068 (PMC13152009; doi:10.1093/jacamr/dlag068)
Supplement: dlag068_Supplementary_Data [file dlag068_supplementary_data.docx]

**Antimicrobial susceptibility trends of WHO critical Gram-negative pathogens in Fijian Hospitals, 2016 - 2021**

**Supplementary**

**Figure S1.0 Map of Fiji**


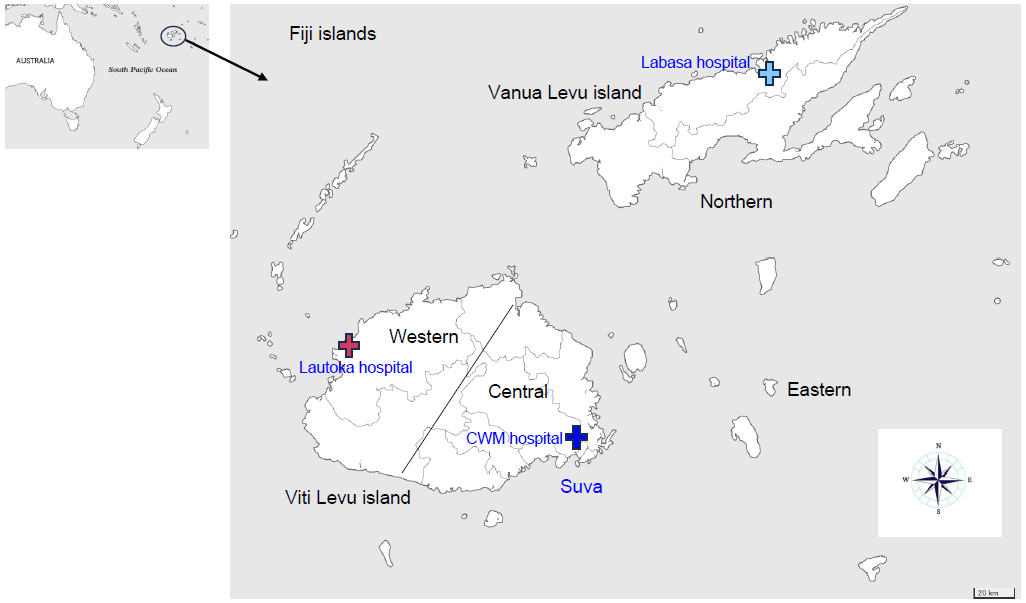


**Map reproduced from Baleivanualala *et. al***^1^

**Clinical microbiology laboratory methods in Fiji.**

**Table S1a Routine culture and biochemical testing**

| **Sample type** | **Preliminary processing** | **Culture medium** | **Incubation temperature** | **O_2_/CO_2_** | **Incubation prior to initial reading of plate** |
| --- | --- | --- | --- | --- | --- |
| Blood^#^ | Gram staining | BA/CA/MC | 37⁰C | Both | 18-24 hours |
| Pus/Wound | Gram staining | PEA/BA/CLED/MC | 37⁰C | O_2_ | 18-24 hours |
| Urine^*^ | Microscopy | BA/CLED/SDA | 37⁰C | O_2_ | 18-24 hours |
| Respiratory | Gram staining | BA/CA/MC/SDA | 37⁰C | Both | 18-24 hours |
| Cerebrospinal fluid | Microscopy | BA/CA/MC | 37⁰C | Both | 18-24 hours |
| Tips and tubes | Subculture on broth | Cooked meat broth, BA/CA/MC | 37⁰C | Both | 18-24 hours |

^#^ Following incubation of blood culture bottles in BacT/ALERT system (bioMérieux, Marcy-Étoile, France).
^*^Urine culture criteria – only those with white blood cells >10 per high powered field, all paediatric cases, all private patients, all cases with renal conditions.

BA, blood agar; CA, chocolate agar; CLED, cystine lactose electrolyte deficient; MC, McConkey agar; SDA, Sabouraud dextrose agar; PEA, phenylethyl alcohol agar.

**Table S1b. Identification methods for key pathogens**

| **Organism** | **Preliminary assessments** | **Biochemical panel** |
| --- | --- | --- |
| *A. baumannii* | Colony morphology | Microbact™ (Oxoid) Gram-negative system biochemical testing systems  OR  Vitek 2 compact (Biomerieux, France) ^#^ |
| *P. aeruginosa* | Colony morphology  Oxidase test (positive) |  |
| *E. coli* | Colony morphology  Indole test (positive) |  |
| *K. pneumoniae* | Colony morphology  Indole test (negative) |  |

^#^ Used at CWMH from 2020 onwards for samples obtained from sterile sites, urine, and other specimens from critical care units.

**Table S1c Fiji antimicrobial susceptibility testing guideline^#¶^**

| **Organism** | **Round 1** | **Round 2** | **Round 3** |
| --- | --- | --- | --- |
| *A. baumannii* | Gentamicin GM (10μg)  Trimethoprim-sulfamethoxazole -SXT (23.75-1.25μg) | Ciprofloxacin – CIP (5μg)  Ceftriaxone - CRO (30μg)  Ceftazidime - CAZ (30μg)  Piperacillin-tazobactam - PIPTAZ (110μg) | Amikacin – AK (30ug)  Meropenem – MEM (10μg) |
| *P. aeruginosa* | Gentamicin -GM (10μg)  Piperacillin -PIP (30μg)  Ciprofloxacin - CIP (5μg) | Piperacillin/tazobactam -PIPTAZ (110μg)  Ceftazidime - CAZ 30μg) | Meropenem - MEM (10μg) |
| Enterobacterales | Ampicillin -AMP (10μg)  Gentamicin – GM (10ug)  Chloramphenicol – CHL (30μg)  Trimethoprim-sulfamethoxazole - SXT (23.75-1.25μg)  Trimethoprim – TMP (5μg) *  Nitrofurantoin - NIT (300μg) *  Cephalothin (30μg) | Ciprofloxacin - CIP(5μg)  Ceftriaxone – CRO (30μg)  Chloramphenicol - CHL (30μg)* | Amikacin – AK (30μg)  Meropenem -MEM (10μg) |
|  |  |  |  |
|  |  | For ESBL testing^§^  Ceftazidime (30μg), ceftazidime +clavulanic acid (30μg+10μg)  Cefotaxime (30μg), cefotaxime +clavulanic acid (30μg+10μg) |  |

^#^**Method**: disk diffusion

^¶^**Culture plate used**: Mueller-Hinton agar made locally using commercial dehydrated media (BD DIFCO Mueller Hinton Agar, Becton Dickinson & Co, Franklin Lakes, NJ, United States of America).

* tested only for isolates from urine samples.

^§^ESBL testing for Enterobacterales was conducted if the organism displayed resistance to gentamicin or resistance to more than three antimicrobials in round 1. Rounds 2 and 3 of testing were contingent upon the availability of antimicrobial disk stock levels, with a focus on prioritizing testing isolates from adult ICU settings.

**Table S1d Internal quality control processes**

| Item | Activities | Frequency |
| --- | --- | --- |
| Culture media | All ATCC strains are cultured on culture media for growth.  Sterility testing – 10% of the prepared culture media should be placed in incubator for determination of sterility | New batch of media  Every batch of prepared media |
| AST | Perform AST using all ATCC strains | Twice a month |
| Gram stain | Perform Gram stain using ATCC strains. | New batch of prepared stain |
| Biochemical testing  e.g. Microbact^TM^, Oxidase | Perform test using ATCC strains | New batch |

All culture and AST medias are made locally using commercial dehydrated media (BD DIFCO, Becton Dickinson & Co, Franklin Lakes, NJ, United States of America). For preparation of BA and PEA, human blood supplied from Fiji blood donor services are being used. Plate used for AST: Mueller Hinton Agar

AST, antimicrobial susceptibility testing; ATCC, American Type Culture Collection.

**Table S1e ATCC strains used for Quality Control**

| **Quality Control Strain** | **ATCC Number** |
| --- | --- |
| *E. coli* | ATCC^®^ 25922 |
| *E. coli* | ATCC^®^ 35218 |
| *K. pneumoniae* | ATCC^®^ 700603 |
| *P. aeruginosa* | ATCC^®^ 27853 |
| *S. aureus* | ATCC^®^ 29213 |

ATCC, American Type Culture Collection

**Table S1f. Quality assurance of CWMH, LTKH, and LBSH microbiology laboratory**

| **PPTC Average score** | | | |
| --- | --- | --- | --- |
|  | **CWMH** | **LTKH** | **LBSH** |
| 2016 |  | 82.5% | 89% |
| 2017 | 89.7% | Not received | 94.50% |
| 2018 | 90.4% | Not received | 91.7% |
| 2019 | 98.2% | Not received | 89% |
| 2020 | 97.8% | Not received | 94% |
| 2021 | 98.5% | 95.0% | 89.33% |

PPTC, Pacific paramedical training centre

**Appendix Table S2a. Aggregated *A. baumannii* susceptibility results by year for all hospitals**

| **Antimicrobial** | **Year** | **Total number tested** | **Susceptible (n)** | **Susceptible (%)** | **Gradient** | **p-value** |
| --- | --- | --- | --- | --- | --- | --- |
| Trimethoprim-sulfamethoxazole | 2016 | 794 | 520 | 65.5% | -1.88 | 0.09 |
|  | 2017 | 835 | 481 | 57.6% |  |  |
|  | 2018 | 993 | 646 | 65.1% |  |  |
|  | 2019 | 1026 | 596 | 58.1% |  |  |
|  | 2020 | 1059 | 582 | 55.0% |  |  |
|  | 2021 | 747 | 413 | 55.3% |  |  |
| Gentamicin | 2016 | 860 | 538 | 62.6% | -1.77 | 0.09 |
|  | 2017 | 960 | 578 | 60.2% |  |  |
|  | 2018 | 1104 | 681 | 61.7% |  |  |
|  | 2019 | 1053 | 544 | 51.7% |  |  |
|  | 2020 | 1083 | 629 | 58.1% |  |  |
|  | 2021 | 881 | 471 | 53.5% |  |  |
| Ciprofloxacin | 2016 | 511 | 332 | 65.0% | -2.67 | 0.01 |
|  | 2017 | 663 | 426 | 64.3% |  |  |
|  | 2018 | 603 | 346 | 57.4% |  |  |
|  | 2019 | 598 | 331 | 55.4% |  |  |
|  | 2020 | 828 | 431 | 52.1% |  |  |
|  | 2021 | 570 | 308 | 54.0% |  |  |
| Ceftriaxone | 2016 | 514 | 183 | 35.6% | -2.04 | 0.13 |
|  | 2017 | 663 | 196 | 29.6% |  |  |
|  | 2018 | 598 | 216 | 36.1% |  |  |
|  | 2019 | 596 | 138 | 23.2% |  |  |
|  | 2020 | 828 | 201 | 24.3% |  |  |
|  | 2021 | 499 | 135 | 27.1% |  |  |
| Ceftazidime | 2020 | 567 | 321 | 56.6% |  | p-value (χ2) |
|  | 2021 | 548 | 276 | 50.4% |  | 0.04 |
| Piperacillin-tazobactam | 2020 | 527 | 314 | 59.6% |  | p-value (χ2) |
|  | 2021 | 454 | 246 | 54.2% |  | 0.09 |
| Amikacin | 2016 | 431 | 233 | 54.1% | -4.58 | 0.007 |
|  | 2017 | 578 | 308 | 53.3% |  |  |
|  | 2018 | 353 | 166 | 47.0% |  |  |
|  | 2019 | 448 | 212 | 47.3% |  |  |
|  | 2020 | 619 | 203 | 32.8% |  |  |
|  | 2021 | 432 | 148 | 34.3% |  |  |
| Meropenem | 2016 | 422 | 255 | 60.4% | -4.25 | 0.0004 |
|  | 2017 | 573 | 327 | 57.1% |  |  |
|  | 2018 | 345 | 178 | 51.6% |  |  |
|  | 2019 | 450 | 221 | 49.1% |  |  |
|  | 2020 | 615 | 252 | 41.0% |  |  |
|  | 2021 | 458 | 187 | 40.8% |  |  |

**Table S2b. Antimicrobial susceptibility of *A. baumannii* by hospital**

| **Antimicrobial** | **Hospital** | **Total number tested** | **Susceptible (n)** | **Resistant (n)** | **Susceptible (%)** | **p-value (χ2)** |
| --- | --- | --- | --- | --- | --- | --- |
| Trimethoprim-sulfamethoxazole | CWMH | 2505 | 1547 | 958 | 61.8% | <0.0001 |
|  | LTKH | 1856 | 997 | 859 | 53.7% |  |
|  | LBSH | 1093 | 694 | 399 | 63.5% |  |
| Gentamicin | CWMH | 2620 | 1589 | 1031 | 60.6% | <0.0001 |
|  | LTKH | 2068 | 1098 | 970 | 53.1% |  |
|  | LBSH | 1253 | 754 | 499 | 60.2% |  |
| Ciprofloxacin | CWMH | 1922 | 1168 | 754 | 60.8% | <0.0001 |
|  | LTKH | 1377 | 791 | 586 | 57.4% |  |
|  | LBSH | 474 | 215 | 259 | 45.4% |  |
| Ceftriaxone | CWMH | 1866 | 478 | 1388 | 25.6% | <0.0001 |
|  | LTKH | 1351 | 475 | 876 | 35.2% |  |
|  | LBSH | 481 | 116 | 365 | 24.1% |  |
| Ceftazidime | CWMH | 457 | 151 | 306 | 33.0% | <0.0001 |
|  | LTKH | 408 | 282 | 126 | 69.1% |  |
|  | LBSH | 250 | 164 | 86 | 65.6% |  |
| Piperacillin-tazobactam | CWMH | 458 | 197 | 261 | 43.0% | <0.0001 |
|  | LTKH | 381 | 246 | 135 | 64.6% |  |
|  | LBSH | 253 | 171 | 82 | 67.6% |  |
| Amikacin | CWMH | 1472 | 739 | 733 | 50.2% | <0.0001 |
|  | LTKH | 981 | 399 | 582 | 40.7% |  |
|  | LBSH | 408 | 132 | 276 | 32.4% |  |
| Meropenem | CWMH | 1480 | 799 | 681 | 54.0% | <0.0001 |
|  | LTKH | 994 | 468 | 526 | 47.1% |  |
|  | LBSH | 389 | 153 | 236 | 39.3% |  |

**Table S2c. Antimicrobial susceptibility of *A. baumannii* by specimen type**

| **Antimicrobial** | **Specimen type** | **Total number tested** | **Susceptible (n)** | **Resistant (n)** | **Susceptible (%)** | **p-value (χ2)** |
| --- | --- | --- | --- | --- | --- | --- |
| Trimethoprim-sulfamethoxazole | Blood | 656 | 357 | 299 | 54.4% | 0.0026 |
|  | Non urine | 4745 | 2876 | 1869 | 60.6% |  |
| Gentamicin | Blood | 588 | 374 | 214 | 63.6% | <0.0001 |
|  | Non urine | 4670 | 2749 | 1921 | 58.9% |  |
|  | Urine | 683 | 318 | 365 | 46.6% |  |
| Ciprofloxacin | Blood | 556 | 347 | 209 | 62.4% | 0.0309 |
|  | Non urine | 2754 | 1573 | 1181 | 57.1% |  |
|  | Urine | 463 | 254 | 209 | 54.9% |  |
| Ceftriaxone | Blood | 550 | 233 | 317 | 42.4% | <0.0001 |
|  | Non urine | 2676 | 711 | 1965 | 26.6% |  |
|  | Urine | 472 | 125 | 347 | 26.5% |  |
| Ceftazidime | Blood | 152 | 68 | 84 | 44.7% | 0.002 |
|  | Non urine | 758 | 433 | 325 | 57.1% |  |
|  | Urine | 205 | 96 | 109 | 46.8% |  |
| Piperacillin-tazobactam | Blood | 119 | 80 | 39 | 67.2% | 0.04 |
|  | Non urine | 770 | 424 | 346 | 55.1% |  |
|  | Urine | 203 | 110 | 93 | 54.2% |  |
| Amikacin | Blood | 363 | 162 | 201 | 44.6% | 0.06 |
|  | Non urine | 2147 | 932 | 1215 | 43.4% |  |
|  | Urine | 351 | 176 | 175 | 50.1% |  |
| Meropenem | Blood | 357 | 189 | 168 | 52.9% | 0.3693 |
|  | Non urine | 2157 | 1056 | 1101 | 49.0% |  |
|  | Urine | 349 | 175 | 174 | 50.1% |  |

**Table S2d. Antimicrobial susceptibility of *A. baumannii* by hospital setting**

| **Antimicrobial** | **Location** | **Total number tested** | **Susceptible (n)** | **Resistant (n)** | **Susceptible (%)** | **p-value (χ2)** |
| --- | --- | --- | --- | --- | --- | --- |
| Trimethoprim-sulfamethoxazole | Inpatient | 4097 | 2368 | 1729 | 57.8% | 0.0002 |
|  | Outpatient | 524 | 339 | 185 | 64.7% |  |
|  | Outer centre | 833 | 531 | 302 | 63.7% |  |
| Gentamicin | Inpatient | 4440 | 2448 | 1992 | 55.1% | <0.0001 |
|  | Outpatient | 615 | 420 | 195 | 68.3% |  |
|  | Outer centre | 886 | 573 | 313 | 64.7% |  |
| Ciprofloxacin | Inpatient | 3084 | 1747 | 1337 | 56.6% | 0.02 |
|  | Outpatient | 246 | 159 | 87 | 64.6% |  |
|  | Outer centre | 443 | 268 | 175 | 60.5% |  |
| Ceftriaxone | Inpatient | 2979 | 770 | 2209 | 25.8% | <0.0001 |
|  | Outpatient | 262 | 100 | 162 | 38.2% |  |
|  | Outer centre | 457 | 199 | 258 | 43.5% |  |
| Ceftazidime | Inpatient | 830 | 423 | 407 | 51.0% | 0.01 |
|  | Outpatient | 89 | 52 | 37 | 58.4% |  |
|  | Outer centre | 196 | 122 | 74 | 62.2% |  |
| Piperacillin-tazobactam | Inpatient | 811 | 438 | 373 | 54.0% | 0.04 |
|  | Outpatient | 94 | 58 | 36 | 61.7% |  |
|  | Outer centre | 187 | 118 | 69 | 63.1% |  |
| Amikacin | Inpatient | 2390 | 1021 | 1369 | 42.7% | 0.0002 |
|  | Outpatient | 180 | 99 | 81 | 55.0% |  |
|  | Outer centre | 291 | 150 | 141 | 51.5% |  |
| Meropenem | Inpatient | 2385 | 1145 | 1240 | 48.0% | 0.0002 |
|  | Outpatient | 181 | 113 | 68 | 62.4% |  |
|  | Outer centre | 297 | 162 | 135 | 54.5% |  |

**Table S3a. Aggregated *P. aeruginosa* susceptibility results by year for all hospitals**

| **Antimicrobial** | **Year** | **Total number tested** | **Susceptible (n)** | **Susceptible (%)** | **Gradient** | **p-value** |
| --- | --- | --- | --- | --- | --- | --- |
| Gentamicin | 2016 | 873 | 663 | 75.9% | -1.26 | 0.002 |
|  | 2017 | 1,056 | 809 | 76.6% |  |  |
|  | 2018 | 1,231 | 916 | 74.4% |  |  |
|  | 2019 | 1,158 | 846 | 73.1% |  |  |
|  | 2020 | 1,383 | 996 | 72.0% |  |  |
|  | 2021 | 931 | 653 | 70.1% |  |  |
| Ceftazidime | 2016 | 869 | 799 | 91.9% | -0.74 | 0.02 |
|  | 2017 | 1,056 | 981 | 92.9% |  |  |
|  | 2018 | 1,232 | 1109 | 90.0% |  |  |
|  | 2019 | 1,162 | 1053 | 90.6% |  |  |
|  | 2020 | 1,113 | 1001 | 89.9% |  |  |
|  | 2021 | 827 | 731 | 88.4% |  |  |
| Piperacillin | 2016 | 874 | 744 | 85.1% | -1.609 | 0.04 |
|  | 2017 | 1,058 | 875 | 82.7% |  |  |
|  | 2018 | 1,179 | 926 | 78.5% |  |  |
|  | 2019 | 469 | 382 | 81.4% |  |  |
|  | 2020 | 295 | 237 | 80.3% |  |  |
|  | 2021 | 249 | 186 | 74.7% |  |  |
| Piperacillin-tazobactam | 2019 | 770 | 712 | 92.5% | -3.55 | 0.157 |
|  | 2020 | 1279 | 1158 | 90.5% |  |  |
|  | 2021 | 541 | 462 | 85.4% |  |  |
| Ciprofloxacin | 2016 | 875 | 604 | 69.0% | -0.38 | 0.69 |
|  | 2017 | 991 | 783 | 79.0% |  |  |
|  | 2018 | 1,222 | 904 | 74.0% |  |  |
|  | 2019 | 1,156 | 842 | 72.8% |  |  |
|  | 2020 | 1,018 | 724 | 71.1% |  |  |
|  | 2021 | 846 | 603 | 71.3% |  |  |
| Meropenem | 2016 | 23 | 23 | 100.0% | -13.87 | 0.005 |
|  | 2017 | 84 | 84 | 100.0% |  |  |
|  | 2018 | 169 | 162 | 95.9% |  |  |
|  | 2019 | 118 | 87 | 73.7% |  |  |
|  | 2020 | 167 | 74 | 44.3% |  |  |
|  | 2021 | 184 | 75 | 40.8% |  |  |

**Table S3b. Antimicrobial susceptibility of *P. aeruginosa* by hospital**

| **Antimicrobial** | **Hospital** | **Total number tested** | **Susceptible (n)** | **Resistant (n)** | **Susceptible (%)** | **p-value (χ2)** |
| --- | --- | --- | --- | --- | --- | --- |
| Gentamicin | CWMH | 2596 | 1968 | 628 | 75.8% | <0.0001 |
|  | LTKH | 2083 | 1595 | 488 | 76.6% |  |
|  | LBSH | 1953 | 1320 | 633 | 67.6% |  |
| Ceftazidime | CWMH | 2416 | 2192 | 224 | 90.7% | 0.0002 |
|  | LTKH | 1972 | 1749 | 223 | 88.7% |  |
|  | LBSH | 1871 | 1733 | 138 | 92.6% |  |
| Piperacillin | CWMH | 1095 | 943 | 152 | 86.1% | <0.0001 |
|  | LTKH | 1323 | 1101 | 222 | 83.2% |  |
|  | LBSH | 1706 | 1306 | 400 | 76.6% |  |
| Piperacillin-tazobactam | CWMH | 1247 | 1116 | 131 | 89.5% | 0.54 |
|  | LTKH | 784 | 713 | 71 | 90.9% |  |
|  | LBSH | 606 | 543 | 63 | 89.6% |  |
| Ciprofloxacin | CWMH | 2290 | 1821 | 469 | 79.5% | <0.0001 |
|  | LTKH | 1928 | 1359 | 569 | 70.5% |  |
|  | LBSH | 1890 | 1281 | 609 | 67.8% |  |
| Meropenem | CWMH | 182 | 98 | 84 | 53.8% | <0.0001 |
|  | LTKH | 196 | 126 | 70 | 64.3% |  |
|  | LBSH | 367 | 281 | 86 | 76.6% |  |

**Table S3c. Antimicrobial susceptibility of *P. aeruginosa* by specimen types**

| **Antimicrobial** | **Specimen type** | **Total number tested** | **Susceptible (n)** | **Resistant (n)** | **Susceptible (%)** | **p-value (χ2)** |
| --- | --- | --- | --- | --- | --- | --- |
| Gentamicin | Blood | 297 | 190 | 107 | 64.0% | <0.0001 |
|  | Non urine | 5532 | 4176 | 1356 | 75.5% |  |
|  | Urine | 803 | 517 | 286 | 64.4% |  |
| Ceftazidime | Blood | 284 | 239 | 45 | 84.2% | <0.0001 |
|  | Non urine | 5266 | 4874 | 392 | 92.6% |  |
|  | Urine | 709 | 561 | 148 | 79.1% |  |
| Piperacillin | Blood | 158 | 122 | 36 | 77.2% | 0.002 |
|  | Non urine | 3590 | 2946 | 644 | 82.1% |  |
|  | Urine | 376 | 282 | 94 | 75.0% |  |
| Piperacillin-tazobactam | Blood | 142 | 123 | 19 | 86.6% | <0.0001 |
|  | Non urine | 2139 | 1974 | 165 | 92.3% |  |
|  | Urine | 356 | 275 | 81 | 77.2% |  |
| Ciprofloxacin | Blood | 287 | 207 | 80 | 72.1% | <0.0001 |
|  | Non urine | 5145 | 3809 | 1336 | 74.0% |  |
|  | Urine | 676 | 445 | 231 | 65.8% |  |
| Meropenem | Blood | 44 | 31 | 13 | 70.5% | 0.48 |
|  | Non urine | 560 | 373 | 187 | 66.6% |  |
|  | Urine | 141 | 101 | 40 | 71.6% |  |

**Table S3d. Antimicrobial susceptibility of *P. aeruginosa* by hospital setting**

| **Antimicrobial** | **Location** | **Total number tested** | **Susceptible (n)** | **Resistant (n)** | **Susceptible (%)** | **p-value (χ2)** |
| --- | --- | --- | --- | --- | --- | --- |
| Gentamicin | Inpatient | 4382 | 3025 | 1357 | 69.0% | <0.0001 |
|  | Outpatient | 1091 | 913 | 178 | 83.7% |  |
|  | Outer centre | 1159 | 945 | 214 | 81.5% |  |
| Ceftazidime | Inpatient | 4193 | 3777 | 416 | 90.1% | 0.06 |
|  | Outpatient | 1014 | 926 | 88 | 91.3% |  |
|  | Outer centre | 1052 | 971 | 81 | 92.3% |  |
| Piperacillin | Inpatient | 2766 | 2184 | 582 | 79.0% | <0.0001 |
|  | Outpatient | 688 | 613 | 75 | 89.1% |  |
|  | Outer centre | 670 | 553 | 117 | 82.5% |  |
| Piperacillin-tazobactam | Inpatient | 1629 | 1429 | 200 | 87.7% | <0.0001 |
|  | Outpatient | 460 | 428 | 32 | 93.0% |  |
|  | Outer centre | 548 | 515 | 33 | 94.0% |  |
| Ciprofloxacin | Inpatient | 3987 | 2618 | 1369 | 65.7% | <0.0001 |
|  | Outpatient | 1038 | 916 | 122 | 88.2% |  |
|  | Outer centre | 1083 | 927 | 156 | 85.6% |  |
| Meropenem | Inpatient | 577 | 380 | 197 | 65.9% | 0.09 |
|  | Outpatient | 90 | 69 | 21 | 76.7% |  |
|  | Outer centre | 78 | 56 | 22 | 71.8% |  |

**Table S4a. Aggregated *E. coli* susceptibility results by year for all hospitals**

| **Antimicrobial** | **Year** | **Total number tested** | **Susceptible (n)** | **Susceptible (%)** | **Gradient** | **p-value** |
| --- | --- | --- | --- | --- | --- | --- |
| Ampicillin | 2016 | 2274 | 612 | 26.9% | -1.83 | 0.06 |
|  | 2017 | 2418 | 553 | 22.9% |  |  |
|  | 2018 | 2339 | 399 | 17.1% |  |  |
|  | 2019 | 3073 | 706 | 23.0% |  |  |
|  | 2020 | 2564 | 445 | 17.4% |  |  |
|  | 2021 | 2267 | 367 | 16.2% |  |  |
| Gentamicin | 2016 | 2274 | 1920 | 84.4% | -3.491 | 0.03 |
|  | 2017 | 2418 | 1916 | 79.2% |  |  |
|  | 2018 | 2339 | 1738 | 74.3% |  |  |
|  | 2019 | 3073 | 2016 | 65.6% |  |  |
|  | 2020 | 2564 | 1916 | 74.7% |  |  |
|  | 2021 | 2267 | 1460 | 64.4% |  |  |
| Trimethoprim-sulfamethoxazole | 2016 | 1311 | 878 | 67.0% | -1.95 | 0.09 |
|  | 2017 | 1379 | 852 | 61.8% |  |  |
|  | 2018 | 1280 | 686 | 53.6% |  |  |
|  | 2019 | 1814 | 1065 | 58.7% |  |  |
|  | 2020 | 1520 | 879 | 57.8% |  |  |
|  | 2021 | 1181 | 646 | 54.7% |  |  |
| Trimethoprim | 2016 | 963 | 486 | 50.5% | -0.64 | 0.09 |
|  | 2017 | 1039 | 545 | 52.5% |  |  |
|  | 2018 | 1059 | 544 | 51.4% |  |  |
|  | 2019 | 1259 | 640 | 50.8% |  |  |
|  | 2020 | 1044 | 521 | 49.9% |  |  |
|  | 2021 | 1089 | 519 | 47.7% |  |  |
| Nitrofurantoin | 2016 | 963 | 896 | 93.0% | -1.869 | 0.01 |
|  | 2017 | 1039 | 959 | 92.3% |  |  |
|  | 2018 | 1059 | 964 | 91.0% |  |  |
|  | 2019 | 1259 | 1144 | 90.9% |  |  |
|  | 2020 | 1044 | 900 | 86.2% |  |  |
|  | 2021 | 1089 | 910 | 83.6% |  |  |
| Chloramphenicol | 2016 | 1572 | 1351 | 85.9% | -4.14 | 0.002 |
|  | 2017 | 1811 | 1509 | 83.3% |  |  |
|  | 2018 | 1737 | 1278 | 73.6% |  |  |
|  | 2019 | 2153 | 1583 | 73.5% |  |  |
|  | 2020 | 1666 | 1186 | 71.2% |  |  |
|  | 2021 | 1296 | 832 | 64.2% |  |  |
| Ciprofloxacin | 2016 | 566 | 391 | 69.1% | -3.409 | 0.0007 |
|  | 2017 | 618 | 415 | 67.2% |  |  |
|  | 2018 | 926 | 578 | 62.4% |  |  |
|  | 2019 | 1037 | 592 | 57.1% |  |  |
|  | 2020 | 889 | 486 | 54.7% |  |  |
|  | 2021 | 1171 | 630 | 53.8% |  |  |
| Cephalothin | 2016 | 1311 | 616 | 47.0 | -4.123 | 0.001 |
|  | 2017 | 1379 | 655 | 47.5 |  |  |
|  | 2018 | 1280 | 520 | 40.6 |  |  |
|  | 2019 | 2280 | 879 | 38.6 |  |  |
|  | 2020 | 1500 | 463 | 30.9 |  |  |
|  | 2021 | 1232 | 351 | 28.5 |  |  |
| Ceftriaxone | 2016 | 566 | 330 | 58.3% | -2.54 | 0.0004 |
|  | 2017 | 618 | 343 | 55.5% |  |  |
|  | 2018 | 909 | 471 | 51.8% |  |  |
|  | 2019 | 1037 | 508 | 49.0% |  |  |
|  | 2020 | 1591 | 777 | 48.8% |  |  |
|  | 2021 | 1604 | 724 | 45.1% |  |  |
| Amikacin | 2016 | 403 | 388 | 96.3% | -1.786 | 0.004 |
|  | 2017 | 396 | 362 | 91.4% |  |  |
|  | 2018 | 433 | 390 | 90.1% |  |  |
|  | 2019 | 660 | 592 | 89.7% |  |  |
|  | 2020 | 844 | 740 | 87.7% |  |  |
|  | 2021 | 711 | 612 | 86.1% |  |  |
| Meropenem | 2016 | 398 | 379 | 95.2% | -2.054 | 0.053 |
|  | 2017 | 396 | 373 | 94.2% |  |  |
|  | 2018 | 447 | 418 | 93.5% |  |  |
|  | 2019 | 660 | 613 | 92.9% |  |  |
|  | 2020 | 862 | 794 | 92.1% |  |  |
|  | 2021 | 849 | 698 | 82.2% |  |  |

**Table S4b. Antimicrobial susceptibility of *E. coli* by hospital**

| **Antimicrobial** | **Hospital** | **Total number tested** | **Susceptible (n)** | **Resistant (n)** | **Susceptible (%)** | **p-value (χ2)** |
| --- | --- | --- | --- | --- | --- | --- |
| Ampicillin | CWMH | 5560 | 1190 | 4370 | 21.4% | <0.0001 |
|  | LTKH | 6364 | 1030 | 5334 | 16.2% |  |
|  | LBSH | 3011 | 860 | 2151 | 28.6% |  |
| Gentamicin | CWMH | 5560 | 4519 | 1041 | 81.3% | <0.0001 |
|  | LTKH | 6364 | 4067 | 2297 | 63.9% |  |
|  | LBSH | 3011 | 2380 | 631 | 79.0% |  |
| Trimethoprim-sulfamethoxazole | CWMH | 3500 | 2047 | 1453 | 58.5% | 0.0002 |
|  | LTKH | 3452 | 2001 | 1451 | 58.0% |  |
|  | LBSH | 1533 | 958 | 575 | 62.5% |  |
| Trimethoprim | CWMH | 2063 | 1238 | 825 | 60.0% | <0.0001 |
|  | LTKH | 2912 | 1342 | 1570 | 46.1% |  |
|  | LBSH | 1478 | 675 | 803 | 45.7% |  |
| Nitrofurantoin | CWMH | 2063 | 1854 | 209 | 89.9% | 0.0002 |
|  | LTKH | 2912 | 2561 | 351 | 87.9% |  |
|  | LBSH | 1478 | 1358 | 120 | 91.9% |  |
| Chloramphenicol | CWMH | 4303 | 3516 | 787 | 81.7% | <0.0001 |
|  | LTKH | 4208 | 2735 | 1473 | 65.0% |  |
|  | LBSH | 1724 | 1488 | 236 | 86.3% |  |
| Ciprofloxacin | CWMH | 2081 | 1299 | 782 | 62.4% | <0.0001 |
|  | LTKH | 2553 | 1415 | 1138 | 55.4% |  |
|  | LBSH | 573 | 378 | 195 | 66.0% |  |
| Cephalothin | CWMH | 3997 | 1699 | 2298 | 42.5% | <0.0001 |
|  | LTKH | 3452 | 947 | 2505 | 27.4% |  |
|  | LBSH | 1533 | 838 | 695 | 54.7% |  |
| Ceftriaxone | CWMH | 2141 | 1079 | 1062 | 50.4% | <0.0001 |
|  | LTKH | 3611 | 1838 | 1773 | 50.9% |  |
|  | LBSH | 573 | 236 | 337 | 41.2% |  |
| Amikacin | CWMH | 1525 | 1489 | 36 | 97.6% | <0.0001 |
|  | LTKH | 1530 | 1262 | 268 | 82.5% |  |
|  | LBSH | 392 | 333 | 59 | 84.9% |  |
| Meropenem | CWMH | 1556 | 1483 | 73 | 95.3% | <0.0001 |
|  | LTKH | 1664 | 1413 | 251 | 84.9% |  |
|  | LBSH | 392 | 379 | 13 | 96.7% |  |

**Table S4c. Antimicrobial susceptibility of *E. coli* by specimen type**

| **Antimicrobial** | **Specimen type** | **Total number tested** | **Susceptible (n)** | **Resistant (n)** | **Susceptible (%)** | **p-value (χ2)** |
| --- | --- | --- | --- | --- | --- | --- |
| Ampicillin | Urine | 6448 | 1260 | 5188 | 19.5% | <0.0001 |
|  | Blood | 2182 | 367 | 1815 | 16.8% |  |
|  | Other | 6305 | 1453 | 4852 | 23.0% |  |
| Gentamicin | Urine | 6448 | 4873 | 1575 | 75.6% | <0.0001 |
|  | Blood | 2182 | 1588 | 594 | 72.8% |  |
|  | Other | 6305 | 4505 | 1800 | 71.5% |  |
| Trimethoprim-sulfamethoxazole | Blood | 2182 | 1105 | 1077 | 50.6% | <0.0001 |
|  | Other | 6303 | 3901 | 2402 | 61.9% |  |
| Trimethoprim | Urine | 6453 | 3255 | 3198 | 50.4% |  |
| Nitrofurantoin | Urine | 6453 | 5773 | 680 | 89.5% |  |
| Chloramphenicol | Urine | 2222 | 1378 | 844 | 62.0% | <0.0001 |
|  | Blood | 2116 | 1781 | 335 | 84.2% |  |
|  | Other | 5897 | 4580 | 1317 | 77.7% |  |
| Ciprofloxacin | Urine | 1521 | 601 | 920 | 39.5% | <0.0001 |
|  | Blood | 1448 | 1138 | 310 | 78.6% |  |
|  | Other | 2238 | 1353 | 885 | 60.5% |  |
| Cephalothin | Blood | 2232 | 730 | 1502 | 32.7% | <0.0001 |
|  | Other | 6750 | 2754 | 3996 | 40.8% |  |
| Ceftriaxone | Urine | 2070 | 778 | 1292 | 37.6% | <0.0001 |
|  | Blood | 1629 | 1104 | 525 | 67.8% |  |
|  | Other | 2626 | 1271 | 1355 | 48.4% |  |
| Amikacin | Urine | 1223 | 1041 | 182 | 85.1% | <0.0001 |
|  | Blood | 644 | 609 | 35 | 94.6% |  |
|  | Other | 1580 | 1434 | 146 | 90.8% |  |
| Meropenem | Urine | 1267 | 1146 | 121 | 90.4% | <0.0001 |
|  | Blood | 646 | 614 | 682 | 95.0% |  |
|  | Other | 1699 | 1515 | 184 | 89.2% |  |

**Table S4d. Antimicrobial susceptibility of *E. coli* by hospital setting**

| **Antimicrobial** | **Location** | **Total number tested** | **Susceptible (n)** | **Resistant (n)** | **Susceptible (%)** | **p-value (χ2)** |
| --- | --- | --- | --- | --- | --- | --- |
| Ampicillin | Inpatient | 9037 | 1667 | 7370 | 18.4% | <0.0001 |
|  | Outpatient | 3016 | 800 | 2216 | 26.5% |  |
|  | Outer centre | 2882 | 613 | 2269 | 21.3% |  |
| Gentamicin | Inpatient | 9037 | 6023 | 3014 | 66.6% | <0.0001 |
|  | Outpatient | 3016 | 2581 | 435 | 85.6% |  |
|  | Outer centre | 2882 | 2362 | 520 | 82.0% |  |
| Trimethoprim-sulfamethoxazole | Inpatient | 5952 | 3329 | 2623 | 55.9% | <0.0001 |
|  | Outpatient | 884 | 598 | 286 | 67.6% |  |
|  | Outer centre | 1649 | 1079 | 570 | 65.4% |  |
| Trimethoprim | Inpatient | 3141 | 1417 | 1724 | 45.1% | <0.0001 |
|  | Outpatient | 2110 | 1177 | 933 | 55.8% |  |
|  | Outer centre | 1202 | 661 | 541 | 55.0% |  |
| Nitrofurantoin | Inpatient | 3141 | 2705 | 436 | 86.1% | <0.0001 |
|  | Outpatient | 2110 | 1966 | 144 | 93.2% |  |
|  | Outer centre | 1202 | 1102 | 100 | 91.7% |  |
| Chloramphenicol | Inpatient | 6898 | 4901 | 1997 | 71.0% | <0.0001 |
|  | Outpatient | 1371 | 1254 | 117 | 91.5% |  |
|  | Outer centre | 1966 | 1584 | 382 | 80.6% |  |
| Ciprofloxacin | Inpatient | 3696 | 2079 | 1617 | 56.3% | <0.0001 |
|  | Outpatient | 582 | 355 | 227 | 61.0% |  |
|  | Outer centre | 929 | 658 | 271 | 70.8% |  |
| Cephalothin | Inpatient | 6313 | 2340 | 3973 | 37.1% | <0.0001 |
|  | Outpatient | 993 | 460 | 533 | 46.3% |  |
|  | Outer centre | 1629 | 684 | 945 | 42.0% |  |
| Ceftriaxone | Inpatient | 4300 | 1866 | 2434 | 43.4% | <0.0001 |
|  | Outpatient | 781 | 452 | 329 | 57.9% |  |
|  | Outer centre | 1244 | 835 | 409 | 67.1% |  |
| Amikacin | Inpatient | 2607 | 2301 | 306 | 88.3% | 0.0001 |
|  | Outpatient | 394 | 372 | 22 | 94.4% |  |
|  | Outer centre | 446 | 411 | 35 | 92.2% |  |
| Meropenem | Inpatient | 2773 | 2471 | 302 | 89.1% | <0.0001 |
|  | Outpatient | 390 | 374 | 16 | 95.9% |  |
|  | Outer centre | 449 | 430 | 19 | 95.8% |  |

**Table S4e. Distribution of *E. coli* ESBL producers**

|  | **Total number of isolates** | **ESBL producer** | **% of ESBLs** | **Gradient** | **p-value** |
| --- | --- | --- | --- | --- | --- |
| 2016 | 2274 | 269 | 11.8% | 2.7 | 0.02 |
| 2017 | 2418 | 258 | 10.7% |  |  |
| 2018 | 2339 | 502 | 21.5% |  |  |
| 2019 | 3073 | 589 | 19.2% |  |  |
| 2020 | 2564 | 513 | 20.0% |  |  |
| 2021 | 2267 | 586 | 25.8% |  |  |
| CWMH | 5560 | 1194 | 21.5% |  |  |
| LTKH | 6364 | 1200 | 18.9% |  |  |
| LBSH | 3011 | 323 | 10.7% |  |  |
| Inpatient | 9037 | 2040 | 22.6% |  |  |
| Outpatient | 3016 | 329 | 10.9% |  |  |
| Outer centre | 2882 | 348 | 12.1% |  |  |
| Urine | 6448 | 1194 | 18.5% |  |  |
| Blood | 2182 | 448 | 20.5% |  |  |
| Non-urine | 6305 | 1075 | 17.0% |  |  |

**ESBL, extended spectrum β-lactamase**

**Table S5a. Aggregated *K. pneumoniae* susceptibility results by year for all hospitals**

| **Antimicrobial** | **Year** | **Total number tested** | **Susceptible (n)** | **Susceptible (%)** | **Gradient** | **p-value** |
| --- | --- | --- | --- | --- | --- | --- |
| Gentamicin | 2016 | 2350 | 1481 | 63.0% | -1.566 | 0.04 |
|  | 2017 | 2694 | 1523 | 56.5% |  |  |
|  | 2018 | 2473 | 1393 | 56.3% |  |  |
|  | 2019 | 2692 | 1444 | 53.6% |  |  |
|  | 2020 | 3966 | 2215 | 55.8% |  |  |
|  | 2021 | 2841 | 1507 | 53.0% |  |  |
| Trimethoprim-sulfamethoxazole | 2016 | 1506 | 1071 | 71.1% | -2.26 | 0.04 |
|  | 2017 | 1908 | 1327 | 69.5% |  |  |
|  | 2018 | 1732 | 1060 | 61.2% |  |  |
|  | 2019 | 1859 | 1110 | 59.7% |  |  |
|  | 2020 | 2222 | 1400 | 63.0% |  |  |
|  | 2021 | 1862 | 1108 | 59.5% |  |  |
| Trimethoprim | 2016 | 844 | 448 | 53.1% | -2.73 | 0.03 |
|  | 2017 | 786 | 409 | 52.0% |  |  |
|  | 2018 | 741 | 299 | 40.4% |  |  |
|  | 2019 | 766 | 342 | 44.6% |  |  |
|  | 2020 | 766 | 312 | 40.7% |  |  |
|  | 2021 | 732 | 292 | 39.9% |  |  |
| Nitrofurantoin | 2016 | 844 | 629 | 74.5% | -2.237 | 0.03 |
|  | 2017 | 786 | 571 | 72.6% |  |  |
|  | 2018 | 741 | 536 | 72.3% |  |  |
|  | 2019 | 766 | 562 | 73.4% |  |  |
|  | 2020 | 766 | 519 | 67.8% |  |  |
|  | 2021 | 732 | 450 | 61.5% |  |  |
| Chloramphenicol | 2016 | 1999 | 1435 | 71.8% | -3.926 | 0.02 |
|  | 2017 | 2311 | 1652 | 71.5% |  |  |
|  | 2018 | 2137 | 1484 | 69.4% |  |  |
|  | 2019 | 2448 | 1621 | 66.2% |  |  |
|  | 2020 | 2781 | 1784 | 64.1% |  |  |
|  | 2021 | 2074 | 1025 | 49.4% |  |  |
| Ciprofloxacin | 2016 | 979 | 687 | 70.2% | -2.074 | 0.002 |
|  | 2017 | 1274 | 829 | 65.1% |  |  |
|  | 2018 | 1264 | 817 | 64.6% |  |  |
|  | 2019 | 1288 | 801 | 62.2% |  |  |
|  | 2020 | 1258 | 757 | 60.2% |  |  |
|  | 2021 | 1375 | 812 | 59.1% |  |  |
| Cephalothin | 2016 | 1506 | 1081 | 71.8% | -5.011 | 0.004 |
|  | 2017 | 1908 | 1172 | 61.4% |  |  |
|  | 2018 | 1724 | 1004 | 58.2% |  |  |
|  | 2019 | 3103 | 1516 | 48.9% |  |  |
|  | 2020 | 2279 | 1179 | 51.7% |  |  |
|  | 2021 | 1862 | 826 | 44.4% |  |  |
| Ceftriaxone | 2016 | 914 | 303 | 33.2% | 0.1657 | 0.69 |
|  | 2017 | 1195 | 366 | 30.6% |  |  |
|  | 2018 | 1254 | 431 | 34.4% |  |  |
|  | 2019 | 1272 | 396 | 31.1% |  |  |
|  | 2020 | 1999 | 666 | 33.3% |  |  |
|  | 2021 | 1521 | 508 | 33.4% |  |  |
| Amikacin | 2016 | 855 | 837 | 97.9% | -0.929 | 0.047 |
|  | 2017 | 1145 | 1087 | 94.9% |  |  |
|  | 2018 | 997 | 935 | 93.8% |  |  |
|  | 2019 | 1118 | 1042 | 93.2% |  |  |
|  | 2020 | 1282 | 1174 | 91.6% |  |  |
|  | 2021 | 1018 | 952 | 93.5% |  |  |
| Meropenem | 2016 | 851 | 835 | 98.1% | -1.134 | 0.549 |
|  | 2017 | 1113 | 1082 | 97.2% |  |  |
|  | 2018 | 984 | 966 | 98.2% |  |  |
|  | 2019 | 1101 | 1089 | 98.9% |  |  |
|  | 2020 | 1448 | 1417 | 97.9% |  |  |
|  | 2021 | 1182 | 1142 | 96.6% |  |  |

**Table S5b. Antimicrobial susceptibility of *K. pneumoniae* by hospital**

| **Antimicrobial** | **Hospital** | **Total number tested** | **Susceptible (n)** | **Resistant (n)** | **Susceptible (%)** | **p-value (χ2)** |
| --- | --- | --- | --- | --- | --- | --- |
| Gentamicin | CWMH | 7131 | 4087 | 3044 | 57.3% | <0.0001 |
|  | LTKH | 5833 | 3069 | 2764 | 52.6% |  |
|  | LBSH | 4052 | 2407 | 1645 | 59.4% |  |
| Trimethoprim-sulfamethoxazole | CWMH | 4579 | 3066 | 1513 | 67.0% | <0.0001 |
|  | LTKH | 3525 | 2047 | 1478 | 58.1% |  |
|  | LBSH | 2985 | 1963 | 1022 | 65.8% |  |
| Trimethoprim | CWMH | 1717 | 811 | 906 | 47.2% | <0.0001 |
|  | LTKH | 1722 | 692 | 1030 | 40.2% |  |
|  | LBSH | 1196 | 599 | 597 | 50.1% |  |
| Nitrofurantoin | CWMH | 1717 | 1144 | 573 | 66.6% | <0.0001 |
|  | LTKH | 1722 | 1219 | 503 | 70.8% |  |
|  | LBSH | 1196 | 904 | 292 | 75.6% |  |
| Chloramphenicol | CWMH | 5774 | 4033 | 1741 | 69.8% | <0.0001 |
|  | LTKH | 4449 | 2477 | 1972 | 55.7% |  |
|  | LBSH | 3527 | 2491 | 1036 | 70.6% |  |
| Ciprofloxacin | CWMH | 2989 | 1954 | 1035 | 65.4% | <0.0001 |
|  | LTKH | 2731 | 1765 | 966 | 64.6% |  |
|  | LBSH | 1718 | 984 | 734 | 57.3% |  |
| Cephalothin | CWMH | 5880 | 3243 | 2637 | 55.2% | <0.0001 |
|  | LTKH | 3525 | 1781 | 1744 | 50.5% |  |
|  | LBSH | 2977 | 1754 | 1223 | 58.9% |  |
| Ceftriaxone | CWMH | 3218 | 855 | 2363 | 26.6% | <0.0001 |
|  | LTKH | 3219 | 1381 | 1838 | 42.9% |  |
|  | LBSH | 1718 | 434 | 1284 | 25.3% |  |
| Amikacin | CWMH | 2884 | 2825 | 59 | 98.0% | <0.0001 |
|  | LTKH | 2074 | 1803 | 271 | 86.9% |  |
|  | LBSH | 1457 | 1399 | 58 | 96.0% |  |
| Meropenem | CWMH | 2940 | 2916 | 24 | 99.2% | <0.0001 |
|  | LTKH | 2282 | 2178 | 104 | 95.4% |  |
|  | LBSH | 1457 | 1437 | 20 | 98.6% |  |

**Table S5c. Antimicrobial susceptibility of *K. pneumoniae* by specimen type**

| **Antimicrobial** | **Specimen type** | **Total number tested** | **Susceptible (n)** | **Resistant (n)** | **Susceptible (%)** | **p-value (χ2)** |
| --- | --- | --- | --- | --- | --- | --- |
| Gentamicin | Blood | 1545 | 823 | 722 | 53.3% | <0.0001 |
|  | Non urine | 10036 | 6278 | 3758 | 62.6% |  |
|  | Urine | 5435 | 2462 | 2973 | 45.3% |  |
| Trimethoprim-sulfamethoxazole | Blood | 1545 | 828 | 717 | 53.6% | <0.0001 |
|  | Non urine | 9544 | 6248 | 3296 | 65.5% |  |
| Trimethoprim | Urine | 4635 | 2102 | 2533 | 45.4% |  |
| Nitrofurantoin | Urine | 4635 | 3267 | 1368 | 70.5% |  |
| Chloramphenicol | Blood | 1490 | 1032 | 458 | 69.3% |  |
|  | Non urine | 9282 | 6873 | 2409 | 74.0% | <0.0001 |
|  | Urine | 2978 | 1096 | 1882 | 36.8% |  |
| Ciprofloxacin | Blood | 1130 | 824 | 306 | 72.9% | <0.0001 |
|  | Non urine | 3793 | 2330 | 1463 | 61.4% |  |
|  | Urine | 2515 | 1549 | 966 | 61.6% |  |
| Cephalothin | Blood | 1711 | 678 | 1033 | 39.6% | <0.0001 |
|  | Other | 10671 | 6100 | 4571 | 57.2% |  |
| Ceftriaxone | Blood | 1210 | 545 | 665 | 45.0% | <0.0001 |
|  | Non urine | 4366 | 1464 | 2902 | 33.5% |  |
|  | Urine | 2579 | 661 | 1918 | 25.6% |  |
| Amikacin | Blood | 753 | 723 | 30 | 96.0% | 0.01 |
|  | Non urine | 3353 | 3130 | 223 | 93.3% |  |
|  | Urine | 2309 | 2174 | 135 | 94.2% |  |
| Meropenem | Blood | 774 | 757 | 17 | 97.8% | <0.0001 |
|  | Non urine | 3523 | 3400 | 682 | 96.5% |  |
|  | Urine | 2382 | 2374 | 8 | 99.7% |  |

**Table S5d. Antimicrobial susceptibility of *K. pneumoniae* by hospital setting**

| **Antimicrobial** | **Location** | **Total number tested** | **Susceptible (n)** | **Resistant (n)** | **Susceptible (%)** | **p-value (χ2)** |
| --- | --- | --- | --- | --- | --- | --- |
| Gentamicin | Inpatient | 11849 | 6035 | 5814 | 50.9% | <0.0001 |
|  | Outpatient | 2482 | 1511 | 971 | 60.9% |  |
|  | Outer centre | 2685 | 2017 | 668 | 75.1% |  |
| Trimethoprim-sulfamethoxazole | Inpatient | 7741 | 4387 | 3354 | 56.7% | <0.0001 |
|  | Outpatient | 1307 | 1045 | 262 | 80.0% |  |
|  | Outer centre | 2041 | 1644 | 397 | 80.5% |  |
| Trimethoprim | Inpatient | 3245 | 1369 | 1876 | 42.2% | <0.0001 |
|  | Outpatient | 837 | 461 | 376 | 55.1% |  |
|  | Outer centre | 553 | 272 | 281 | 49.2% |  |
| Nitrofurantoin | Inpatient | 3245 | 2281 | 964 | 70.3% | 0.5239 |
|  | Outpatient | 837 | 585 | 252 | 69.9% |  |
|  | Outer centre | 553 | 401 | 152 | 72.5% |  |
| Chloramphenicol | Inpatient | 9801 | 6053 | 3748 | 61.8% | <0.0001 |
|  | Outpatient | 1687 | 1217 | 470 | 72.1% |  |
|  | Outer centre | 2262 | 1731 | 531 | 76.5% |  |
| Ciprofloxacin | Inpatient | 5942 | 3730 | 2212 | 62.8% | 0.0057 |
|  | Outpatient | 690 | 423 | 267 | 61.3% |  |
|  | Outer centre | 806 | 550 | 256 | 68.2% |  |
| Cephalothin | Inpatient | 8721 | 4090 | 4631 | 46.9% | <0.0001 |
|  | Outpatient | 1369 | 1017 | 352 | 74.3% |  |
|  | Outer centre | 2292 | 1671 | 621 | 72.9% |  |
| Ceftriaxone | Inpatient | 6216 | 1803 | 4413 | 29.0% | <0.0001 |
|  | Outpatient | 837 | 302 | 535 | 36.1% |  |
|  | Outer centre | 1102 | 565 | 537 | 51.3% |  |
| Amikacin | Inpatient | 5261 | 4929 | 332 | 93.7% | 0.155 |
|  | Outpatient | 582 | 552 | 30 | 94.8% |  |
|  | Outer centre | 572 | 546 | 26 | 95.5% |  |
| Meropenem | Inpatient | 5463 | 5334 | 129 | 97.6% | 0.23 |
|  | Outpatient | 634 | 624 | 10 | 98.4% |  |
|  | Outer centre | 582 | 573 | 9 | 98.5% |  |

**Table S5e. Distribution of *K. pneumoniae* ESBL producers**

|  | **Total number of isolates** | **ESBL producer** | **% of ESBLs** | **Gradient** | **p-value** |
| --- | --- | --- | --- | --- | --- |
| 2016 | 2350 | 775 | 33.0% | -0.39 | 0.83 |
| 2017 | 2694 | 1083 | 40.2% |  |  |
| 2018 | 2473 | 1009 | 40.8% |  |  |
| 2019 | 2692 | 1311 | 48.7% |  |  |
| 2020 | 3966 | 1251 | 31.5% |  |  |
| 2021 | 2841 | 964 | 33.9% |  |  |
| CWMH | 7131 | 2708 | 38.0% |  |  |
| LTKH | 5833 | 2114 | 36.2% |  |  |
| LBSH | 4052 | 1571 | 38.8% |  |  |
| Inpatient | 11849 | 5262 | 44.4% |  |  |
| Outpatient | 2482 | 579 | 23.3% |  |  |
| Outer centre | 2685 | 552 | 20.6% |  |  |
| Urine | 5435 | 2443 | 44.9% |  |  |
| Blood | 1545 | 750 | 48.5% |  |  |
| Other | 10036 | 3200 | 31.9% |  |  |

**Reference**

1. Baleivanualala SC, Matanitobua S, Soqo V, *et al.* Molecular and clinical epidemiology of carbapenem resistant Acinetobacter baumannii, Pseudomonas aeruginosa and Enterobacterales in Fiji: a multicentre prospective observational study. *The Lancet Regional Health - Western Pacific* 2024; **47**: 101095.
